# The Bioavailability, Biodistribution, and Toxic Effects of Silica-Coated Upconversion Nanoparticles *in vivo*

**DOI:** 10.3389/fchem.2019.00218

**Published:** 2019-04-10

**Authors:** Mingzhu Zhou, Xiaoqian Ge, Da-Ming Ke, Huan Tang, Jun-Zheng Zhang, Matteo Calvaresi, Bin Gao, Lining Sun, Qianqian Su, Haifang Wang

**Affiliations:** ^1^Institute of Nanochemistry and Nanobiology, Shanghai University, Shanghai, China; ^2^Research Center of Nano Science and Technology, and School of Material Science and Engineering, Shanghai University, Shanghai, China; ^3^Beijing National Laboratory for Molecular Sciences, College of Chemistry and Molecular Engineering, Peking University, Beijing, China; ^4^Dipartimento di Chimica “G. Ciamician,” Alma Mater Studiorum–Università di Bologna, Bologna, Italy; ^5^Cancer and Stem Cell Biology Program, Duke-NUS Medical School, Singapore, Singapore

**Keywords:** upconversion nanoparticle, bioavailability, distribution, toxicity, *in vivo*, gavage

## Abstract

Lanthanide-doped upconversion nanoparticles can convert long wavelength excitation radiation to short wavelength emission. They have great potential in biomedical applications, such as bioimaging, biodetection, drug delivery, and theranostics. However, there is little information available on their bioavailability and biological effects after oral administration. In this study, we systematically investigated the bioavailability, biodistribution, and toxicity of silica-coated upconversion nanoparticles administrated by gavage. Our results demonstrate that these nanoparticles can permeate intestinal barrier and enter blood circulation by microstructure observation of Peyer's patch in the intestine. Comparing the bioavailability and the biodistribution of silica-coated upconversion nanoparticles with oral and intravenous administration routes, we found that the bioavailability and biodistribution are particularly dependent on the administration routes. After consecutive gavage for 14 days, the body weight, pathology, Zn and Cu level, serum biochemical analysis, oxidative stress, and inflammatory cytokines were studied to further evaluate the potential toxicity of the silica-coated upconversion nanoparticles. The results suggest that these nanoparticles do not show overt toxicity in mice even at a high dose of 100 mg/kg body weight.

## Introduction

In the last decade, lanthanide-doped upconversion nanoparticles have attracted increasing attention because of their unique advantages, such as low level of background noise, deep penetration depth, minimal photodamage, and high resistance to photobleaching (Auzel, 2004; Lu et al., 2013; Bettinelli et al., 2015; Li et al., 2015b, 2017; Jalani et al., 2018; Liu et al., 2018; Sun et al., 2018). Surface modification is typically required to make upconversion nanoparticles, UCNPs, suitable for biomedical application, which typically involves coating a hydrophilic ligand (i.e., amphiphilic polymers, proteins) or an extra hydrophilic layer (i.e., SiO_2_) on their surface (Li et al., 2015a; Liu et al., 2015; Sedlmeiera and Gorris, 2015; Plohl et al., 2017). These features made UCNPs suitable for many biological and medical applications, including multimodal bioimaging, biosensing, drug delivery, photodynamic therapy, and synergetic therapy (Lim et al., 2006; Liu et al., 2012, 2016; Yang et al., 2015; Zhou et al., 2015; Su et al., 2017; Chen et al., 2018; Green et al., 2018; Tsai et al., 2018). Despite the encouraging results that have been obtained, there are many unresolved issues relating to the biological effects of these nanomaterials.

UCNPs acting as drug carriers, contrast agents, or bioprobes have been extensively studied either in mice or in plant models (Peng et al., 2012; Liu et al., 2013; Chen et al., 2014; Wu et al., 2016). In previous studies, the majority of UCNPs toxicity assays were performed on different cell lines *in vitro* (Gnach et al., 2015; Tian et al., 2015; Wozniak et al., 2016; Wysokinska et al., 2016; Gao et al., 2017), but fewer reports focused on *in vivo* toxicity studies (Cheng et al., 2011; Wang et al., 2013; Jang et al., 2014; Lucky et al., 2016). The toxicity assays of UCNPs were routinely carried out based on the intravenous injection technique (Abdul and Zhang, 2008; Xiong et al., 2010; Zhou et al., 2011; Ramirez-Garcia et al., 2017). Very recently, Ortgies et al. developed an orally administrated lanthanide-doped UCNP for multiplexed imaging and drug delivery (Ortgies et al., 2018). It is also worth noting that oral administration of substances is a common route in scientific experiments using small animals, such as mice. However, a comprehensive study of the biodistribution and toxicity of UCNPs undergoing oral administration route was not found. Furthermore, since nanoparticles have larger sizes compared to conventional drugs, UCNPs can be poorly absorbed via the oral route. For this reason, it is important to examine whether these nanoparticles can permeate epithelial barriers, in particular the intestinal barrier. There is little information available about the bioavailability of these nanoparticles through oral exposure. Therefore, it is necessary to assess the bioavailability, distribution, and toxicity of UCNPs administrated orally.

In this study, a systematic investigation of the bioavailability, biodistribution, and toxicity of orally administered silica-coated NaYF_4_:Yb,Er nanoparticles (NaYF_4_:Yb,Er@SiO_2_) with an average diameter of 50 nm was carried out in mice. NaYF_4_:Yb,Er@SiO_2_ nanoparticles are chosen because of their good biocompatibility, broad bioapplications, and suppression of lanthanide leakage (Liu et al., 2015). We envision that NaYF_4_:Yb,Er@SiO_2_ nanoparticles can be absorbed though Peyer's patch in intestine and then enter the blood circulation of mice. We also compare the biodistribution of orally administrated NaYF_4_:Yb,Er@SiO_2_ with that of intravenously administrated NaYF_4_:Yb,Er@SiO_2_ by TEM and inductively coupled plasma mass spectrometry (ICP-MS). The toxicity of NaYF_4_:Yb,Er@SiO_2_ is determined by several different approaches, including body weight measurement, pathology changes observation, Zn and Cu levels, serum biochemical analyses, oxidative stress, and inflammatory cytokines analysis.

## Materials and Methods

### Materials

Yttrium(III) chloride hexahydrate (99.9%), ytterbium(III) chloride hexahydrate (99.9%), erbium(III) chloride hexahydrate (99.9%), oleic acid (technical grade, 90%), 1-octadecene (technical grade, 90%), Igepal CO-520 and tetraethyl orthosilicate (TEOS, 99.0%) were purchased from Sigma Aldrich. Sodium hydroxide (96%), ammonium fluoride (98%), methanol (99.5%), and ammonia solution (25–28%) were obtained from Aladdin. Nitric acid (CMOS), hydrofluoric acid (guaranteed grade), and perchloric acid (guaranteed grade) were purchased from Sinopharm Chemical Reagent Co., Ltd., Shanghai, China. All chemicals were used as received without further purification.

### Characterization

The size and morphology of the nanoparticles were characterized on a low-to-high resolution transmission electron microscope (JEM-2010F, JEOL, Japan) operated at 120 kV. Powder X-ray diffraction (XRD, Nano 90ZS, Malven, Britain) measurement was performed on a 3 kW D/MAX2200 V PC diffractometer using Cu kα radiation (60 kV, 80 mA) at a step width of 8° min^−1^. Fourier transform infrared spectroscopy (FT-IR) spectra were obtained in the spectral range from 4,000 to 400 cm^−1^ on an Avatar 370 (Nicolet, America) instrument using the pressed KBr pellet technique. The microstructure observation of Peyer's patch and liver tissue was conducted on a transmission electron microscopy (JEM-1200EX, JEOL, Japan). All biochemical assays were performed using a Hitachi 7,080 clinical automatic chemistry analyzer (Japan).

### Synthesis of NaYF_4_:Yb,Er Upconversion Nanoparticles

In a typical experiment, YCl_3_ (1.56 mmol, 78%), YbCl_3_ (0.4 mmol, 20%), and ErCl_3_ (0.04 mmol, 2%) dissolved in deionized water were added into a 100 mL flask. The solution was then heated to 110°C to evaporate water until the solution became white powder. Subsequently, 12 mL oleic acid and 30 mL 1-octadecene were added in the mixture. The mixture was then heated to 150°C and kept at this temperature for 1 h before cooling down to 50°C. Twenty milliliters of methanol solution containing NaOH (0.2 g, 1.6 mmol) and NH_4_F (0.3 g, 8 mmol) was added into the flask and stirred for 30 min at 100°C to evaporate methanol. After that, the mixture was heated to 300°C and kept for 1 h under nitrogen atmosphere. The obtained mixture was precipitated by the addition of acetone, separated by centrifugation, and washed with cyclohexane. The resulting nanoparticles NaYF_4_:Yb,Er were redispersed in 20 mL cyclohexane.

### Synthesis of NaYF_4_:Yb,Er@SiO_2_ Nanoparticles

Igepal CO-520 (1 mL) was dispersed in 20 mL of cyclohexane, and then a 1.5 mL NaYF_4_:Yb,Er nanoparticle in cyclohexane solution was added into the mixture. After stirring for 3 h, 200 μL TEOS was added and the mixture continue stirred 0.5 h. Then, 130 μL ammonia was injected into the mixture and the mixture was sealed and kept stirring for 20 h. The product was precipitated using methanol, collected by centrifugation, and washed with ethanol several times. Finally, the product was dispersed in deionized water.

### Stability Assay

To determine the stability of NaYF_4_:Yb,Er@SiO_2_ in physiological solutions, the dissolutions of NaYF_4_:Yb,Er@SiO_2_ nanoparticles were monitored in fetal bovine serum (FBS) and simulated body fluid (SBF). SBF was prepared following the recipe in Table S1 (Tadashi and Hiroaki, 2006). NaYF_4_:Yb/Er@SiO_2_ (0.1 mL) at a concentration of 20 mg/mL was mixed with 0.9 mL of FBS or SBF in a glass bottle. The mixture was incubated in a thermostatic shaker (150 rpm) at 37°C. After incubation for predetermined period of time, the resulting solution was centrifuged at 12,000 rpm for 15 min. The supernatant was collected and digested with 1 mL 70% HNO_3_ and 0.5 mL 30% H_2_O_2_ at 90°C. When the solution became clear and colorless, the solution was adjusted to 8 mL by using 2% HNO_3_. The content of yttrium (Y) in the solution was measured by inductively coupled plasma mass spectrometry (ICP-MS, ELAN DRC-e, PerkinElmer Co., Ltd., USA).

### *In vivo* Experiment

All animal experiments were carried out in accordance with the guide for the animal care and use program guidelines of Shanghai University with the approval by Shanghai University. In this study, 60 mice were divided into gavage (36 mice) and intravenous administration (24 mice) groups. Six-week-old healthy male ICR mice (22–26 g) were supplied by the Experimental Animal Center, Second Military Medical University (Shanghai, China). The mice were housed in clean polypropylene cages (6 mice/cage) with the commercial pellet diet and water *ad libitum* at 22 ± 2°C and kept on a 12 h light/dark cycle. After acclimation for 1 week, four groups of mice were treated with NaYF_4_:Yb/Er@SiO_2_ (treatment group, *n* = 6) daily by gavage for 7 and 14 consecutive days with doses of 20 mg/kg bodyweight (b.w.) and 100 mg/kg (b.w.). The two corresponding control groups (*n* = 6) were treated with water daily by gavage for 7 and 14 consecutive days. In addition, four groups of mice were intravenously injected with NaYF_4_:Yb/Er@SiO_2_ (treatment group, 20 mg/kg b.w, *n* = 6) and saline (control group, *n* = 6) through mouse tail veins and sacrificed at 1 and 7 days after injection. All mice were weighed daily during the experimental period. And all experiments were repeated twice.

### Biodistribution of Yttrium, Zinc, and Copper in Mice

At predetermined time points, mice were sacrificed and the contents of Y, Zn, and Cu in main organs were measured after consecutive gavage administration of NaYF_4_:Yb/Er@SiO_2_. Liver, kidneys, spleen, lungs, heart, bone, stomach, large intestine, and small intestine were collected and about 0.1–0.3 g of these organs and were digested with 70% nitric acid and hydrofluoric acid by microwave digestion system (MARS, USA). In addition, the blood samples were digested by the same method as organs. Then, perchloric acid was added into the digested solution and the mixture was heated at 200°C to remove the remaining nitric acid and hydrofluoric acid. When water was evaporated, ultrapure water was added twice. The resulting solution was adjusted to 8 mL with 2% nitric acid solution and the metal content in solution was determined by ICP-MS.

### Microstructure Observation of Peyer's Patch and Liver Tissue

Peyer's patch and liver tissue (1 mm cubes) were collected from small intestine and liver, respectively. They were subsequently fixed in 2.5% glutaraldehyde in phosphate buffer for 2 h and rinsed by 0.1 M phosphate rinsing fluid for three times. After that, the samples were post-fixed with 1% osmium tetraoxide at 4°C for 2 h and then dehydrated with ethanol and acetone as follows, 50% ethanol−15 min, 70% ethanol−15 min, 80% ethanol−15 min, 90% ethanol−15 min, 100% ethanol−20 min, and 100% acetone−20 min. Then the tissue blocks were infiltrated with embedding medium in acetone (v:v = 1:1) for 3 h, and infiltrated with embedding medium overnight. The embedded tissue blocks in embedding medium were polymerized in a dry centrifuge tube at 70°C overnight. The blocks were then cut in 50–70 nm thickness using an ultramicrotome (LKB-I, Sweden), and then counterstained with 3% uranyl acetate and lead citrate prior to TEM measurements.

### Organ Index

Organ indices were calculated for major organs using the following formula: (organ weight)/(total body weight) × 100.

### Histopathological Investigation

The organs including liver, kidney, lung, spleen, and small intestine were collected at each time point and fixed with formalin. The fixed organs were embedded in paraffin, sliced at a thickness of 5 μm and then placed onto glass slides. After hematoxylin–eosin (H&E) staining, the slides were investigated and photographed on an optical microscope (DM750, Leica, Germany).

### Serum Biochemistry Analysis

Blood samples were collected from mice at predetermined time point and centrifuged at 3,000 rpm for 15 min. The obtained serum samples were stored at −20°C before analysis. Alanine aminotransferase (ALT), aspartate aminotransferase (AST), alkaline phosphatase (ALP), blood urea nitrogen (BUN), and creatinine (CREA) were measured using the commercial kits (The Seno Clinical Diagnostic Products Co., Japan).

### Oxidative Stress Assay

The organs including liver, lung, kidney and spleen were rinsed with saline solution at 4°C and then wiped with dry filter papers. Ten or Five percent (w/v) homogenates were prepared by homogenization of tissue in saline solution at 10,000 rpm for 3 min using a homogenizer. The supernatants were collected after centrifuging the homogenates at 3,500 rpm for 10 min. The contents of protein in the supernatants were examined by bicinchoninic acid assay (BCA protein assay kit, Nanjing Jiancheng bioengineering institute, Nanjing, China). The reduced GSH levels of the supernatants were determined by Ellman's reagent 5,5'dithiobis-(2-nitrobenzoic acid) (DTNB, Nanjing Jiancheng Biotechnology Institute, China). The lipid peroxidation indicator malondialdehyde (MDA) was estimated by the method of thiobarbituric acid reactive species (TBA, Nanjing Jiancheng Bioengineering Institute, Nanjing, China).

### TNF-α, IL-6, and IL-1β Levels in Liver

The contents of TNF-α, IL-6, and IL-1β in supernatants of liver homogenates were quantified by a double-antibody sandwich ELISA commercial kit (BD Biosciences, USA). Manufacturer's protocol was followed. The absorbance was measured on a microplate reader at 450 nm (Varioskan Flash, Thermo, USA) and the contents of TNF-α, IL-6, and IL-1β were calculated based on the corresponding standard curves. The protein contents in supernatants were also determined by bicinchoninic acid assay (BCA protein assay kit, Nanjing Jiancheng bioengineering institute, Nanjing, China). The levels of TNF-α, IL-6, and IL-1β were expressed as ng/mg protein.

### Statistical Analysis

All data were expressed as the mean ± standard deviation (mean ± SD) of more than three individual observations. Significance was calculated using Student's *t*-test. The difference was considered significant if *p* < 0.05. In addition, standard deviation and *p*-value were calculated by:

$$\begin{array}{l} \text{Standard deviation }(\text{SD})=\sqrt{\frac{\sum {\left(\text{X-M}\right)}^{2}}{n-1}},\\ \;S=\sqrt{\frac{\left(n_{1}-1\right)SD_{1}^{2}+\left(n_{2}-1\right)SD_{2}^{2}}{n_{1}+n_{2}-2}},\\ \;\text{p}=\frac{\left|M_{1}-M_{2}\right|}{S}\sqrt{\frac{n_{1}n_{2}}{n_{1}+n_{2}}} \end{array}$$

*X* represents the data value, *M* refers to the average value between the data, *S* represents the pooled estimate of the standard deviation, *n* represents the number of data (Gardner and Altman, 1986).

## Results and Discussion

### Characterization of NaYF_4_:Yb,Er@SiO_2_

**Figure 1:**
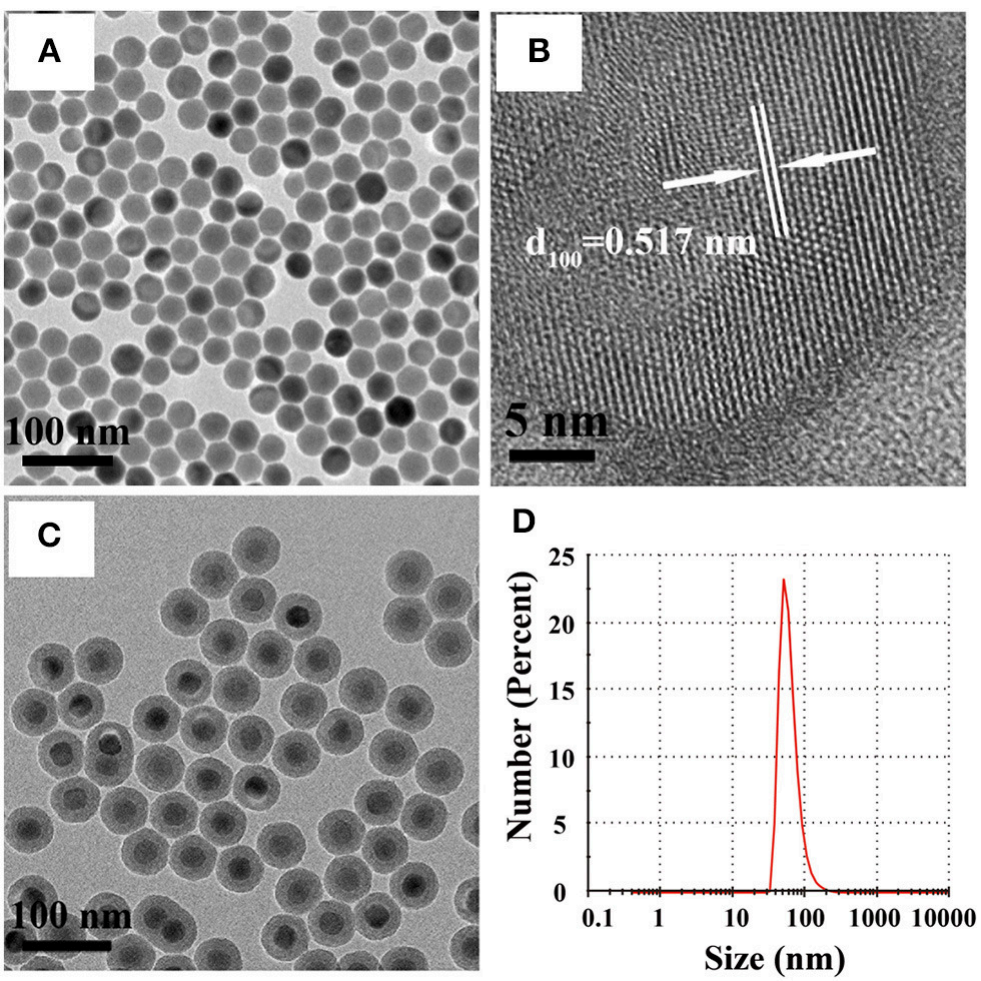
**(A,B)** TEM and HRTEM images of NaYF_4_:Yb,Er nanoparticles in cyclohexane. **(C)** TEM image of NaYF_4_:Yb,Er@SiO_2_ nanoparticles in water. **(D)** Dynamic light scattering (DLS) of NaYF_4_:Yb,Er@SiO_2_ nanoparticles in water.

We first characterized the morphology and size of NaYF_4_:Yb,Er nanoparticles using a transmission electron microscope (TEM). As shown in Figure 1A and Figure S1A, uniform nanoparticles with an average diameter of around 32 nm were obtained. The obtained nanoparticles were confirmed to be single crystals with a hexagonal phase by high-resolution transmission electron microscopy (HRTEM) and X-ray powder diffraction (XRD) study (Figure 1B and Figure S2). The lattice distance of 0.517 nm corresponds to the *d* spacing for (100) plane of hexagonal NaYF_4_. After coating with a silica layer on the surface of NaYF_4_:Yb,Er nanoparticles, the size of the nanoparticles reached 49 nm (Figure 1C and Figure S1B). The results of dynamic light scattering (DLS) measurement show that the hydrodiameter of NaYF_4_:Yb,Er@SiO_2_ nanoparticles was around 60 nm (PDI = 0.23), confirming their mono-dispersion in aqueous solution (Figure 1D). The XRD pattern of NaYF_4_:Yb/Er samples can also be indexed as a hexagonal phases of NaYF_4_ (JCPDS file number 16-0334) (Figure S1). After silica coating, a broad diffraction peak at 2θ = 22° appeared, which can be ascribed to the peak of amorphous silica. In addition, the presence of the elements (Si, O, F, Y, Yb, Er) in the energy dispersive X-ray (EDX) spectrum also confirmed that silica shell was successfully coated onto the surface of NaYF_4_:Yb/Er nanoparticles (Figure S3).

FT-IR spectra of oleic acid coated NaYF_4_:Yb,Er and NaYF_4_:Yb,Er@SiO_2_ nanoparticles were shown in Figure S4. In the spectrum of oleic acid coated NaYF_4_:Yb,Er, the peaks at 2,927 and 2,857 cm^−1^ are attributed to the asymmetric and symmetric stretching vibration of methylene (CH_2_) in the long alkyl chain of oleic acid, and the 1,557 and 1,460 cm^−1^ bands are assigned to the asymmetric and symmetric stretching vibration of the carboxylic group (–COOH) in oleic acid. In the spectrum of NaYF_4_:Yb,Er@SiO_2_, the bands of Si–O–Si are located at 1,091 and 799 cm^−1^, and the peaks at 953, 1,637, and 3,428 cm^−1^ are assigned to Si–OH, H_2_O, and –OH.

### Stability Assay of NaYF_4_:Yb,Er@SiO_2_ in Physiological Solution

In order to study the stability of NaYF_4_:Yb,Er@SiO_2_ under physiological conditions, the dissolved yttrium ions (Y^3+^) of NaYF_4_:Yb,Er@SiO_2_ in FBS and SBF was monitored by ICP-MS. As shown in Figure S5, while the percentage of dissolved Y^3+^ in SBF was only around 0.1%, in FBS it quickly increased to around 1.2%. This result was consistent with the dissolution of Ag_2_Se quantum dots, which was about 4–5% in FBS and <1% in SBF (Tang et al., 2016). Compared with naked UCNPs, these results imply that silica coating can suppress the leakage of lanthanide from nanoparticles, offering an excellent stability of NaYF_4_:Yb,Er@SiO_2_ under physiological conditions (Wang et al., 2012; Tian et al., 2015).

### Stability of NaYF_4_:Yb,Er@SiO_2_ After *in vivo* Gastrointestinal Digestion

To examine the stability of NaYF_4_:Yb,Er@SiO_2_ after *in vivo* gastrointestinal digestion, we investigated the morphology and size of NaYF_4_:Yb,Er@SiO_2_ collected in feces excreted by mice orally administrated NaYF_4_:Yb,Er@SiO_2_ (100 mg/kg b.w.) by using TEM image. As shown in Figure S6, the morphology and size of NaYF_4_:Yb,Er@SiO_2_ was essentially not changed. This result suggest that SiO_2_ coated UCNPs are also stable in the low gastric pH, which is consistent with the previous results of silica coated quantum dots (Loginova et al., 2012). This result also indicates that UCNPs with silica coating can be used to visualize the gastrointestinal tract *in vivo*.

### Biodistribution of NaYF_4_:Yb,Er@SiO_2_

**Figure 2:**
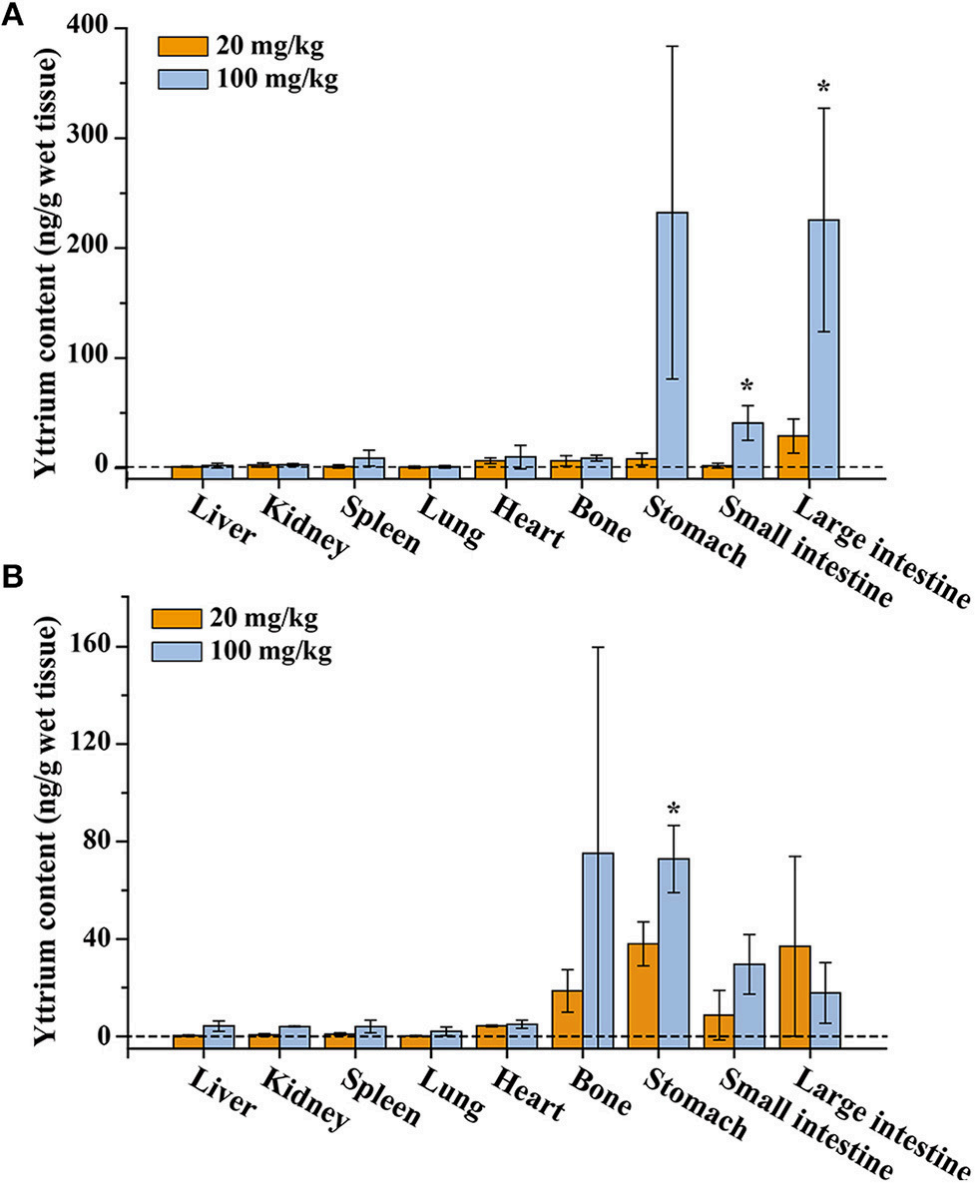
Yttrium content in tissues of the mice after consecutive gavage administration of NaYF_4_:Yb,Er@SiO_2_ for 7 days **(A)** and 14 days **(B)**. *Significant difference between the two experimental groups (*P* < 0.05, *n* = 3).

For the oral administration group, a relatively high concentration of Y^3+^ was detected in the gastrointestinal tract after 7 and 14 days consecutive oral exposure (Figure 2). In contrast, relatively low Y^3+^ concentrations were detected in several other major organs including liver, kidney, spleen, lung, heart, and bone. The concentration of yttrium in all organs increased to a relatively higher level at a high dose of 100 mg/kg compared with the dose of 20 mg/kg. Note that very little amount of Y^3+^ was released from NaYF_4_:Yb,Er@SiO_2_ as mentioned above, thus, it is reasonable to deduce that the Y^3+^ ion in the organs did not come from released ions of NaYF_4_:Yb,Er@SiO_2_. This may be attributed to the fact that after passing through the mouth and stomach, a small amount of NaYF_4_:Yb,Er@SiO_2_ are absorbed by the epithelium of the digestive tract and enter the blood, subsequently resulting in the accumulation of these particles in the organs.

Unlike oral administration, a very high concentration of Y^3+^ was mainly accumulated in the liver of mice after intravenous injection of these nanoparticles. With the lapse of time, the concentration of Y^3+^ decreased in liver and increased in spleen, indicating these nanoparticles follow a hepatic metabolic pathway (Figure S7). This result is also consistent with previous reports (Yu et al., 2017). In addition, we also examined the content of ytterbium ion (Yb^3+^) in organs (Figure S8). We found that the change of Yb^3+^ content was the same as that of Y^3+^, further indicating the good stability of those silica-coated upconversion nanoparticles *in vivo*.

### Ultramicrostructure Observation of Peyer's Patch and Liver Tissue

**Figure 3:**
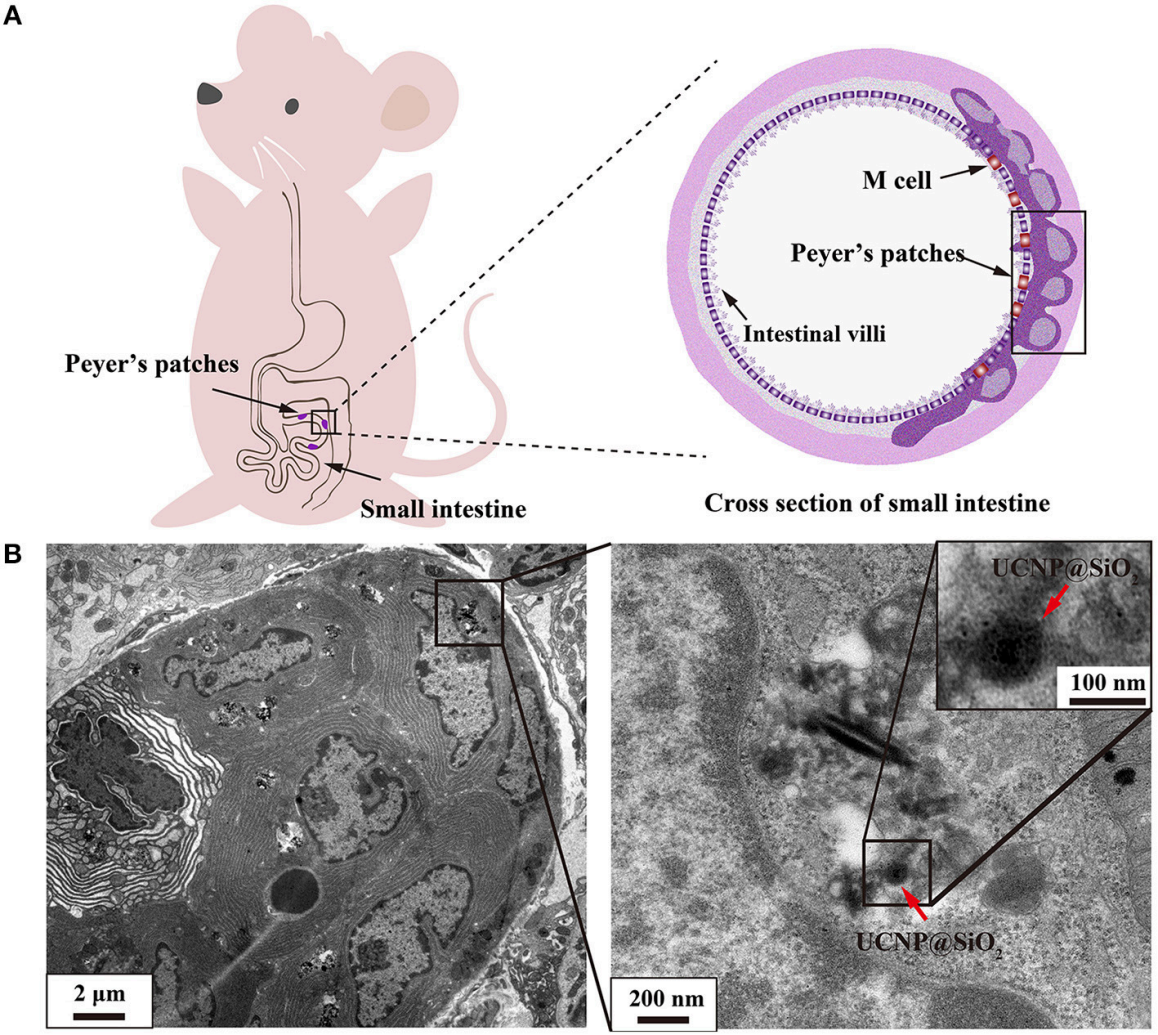
**(A)** Schematic illustration of Peyer's patches in small intestine. **(B)** TEM image demonstrated that NaYF_4_:Yb,Er@SiO_2_ nanoparticles were located in Peyer's patch in small intestine of mice after 14 days' consecutive gavage administration of these nanoparticles. UCNP@SiO_2_ denotes NaYF_4_:Yb,Er@SiO_2_.

To identify the potential uptake mechanism, we carefully examined the samples of small intestine after 14-day consecutive gavage of NaYF_4_:Yb,Er@SiO_2_ at a dose of 100 mg/kg by TEM image. It was reported that large size particles are probably absorbed by Peyer's patches through microfold (M) cells (Lundquist and Artursson, 2016). Peyer's patches are located in the mucous membrane lining of the intestine. They play a role in immunologic response (Figure 3A). Several studies have tested the translocation of nanoparticles in cell models (Yoshida et al., 2014; Walczak et al., 2015; Yao et al., 2015; Chen et al., 2016). However, there is no report to demonstrate this assumption *in vivo*. Here, we utilized TEM to visualize the location of the nanoparticles. As shown in Figure 3B, it can be clearly observed that nanoparticles are located in Peyer's patches in small intestine of mice. The size and shape of these nanoparticles were the same as those we synthetized. This result confirms that the nanoparticles can cross small intestine through the uptake by Peyer's patches. However, due to the limited number of M cells in Peyer's patches, who compose <1 percent of the small intestine epithelial cell layer, we speculate that the bioavailability of these nanoparticles is low.

**Figure 4:**
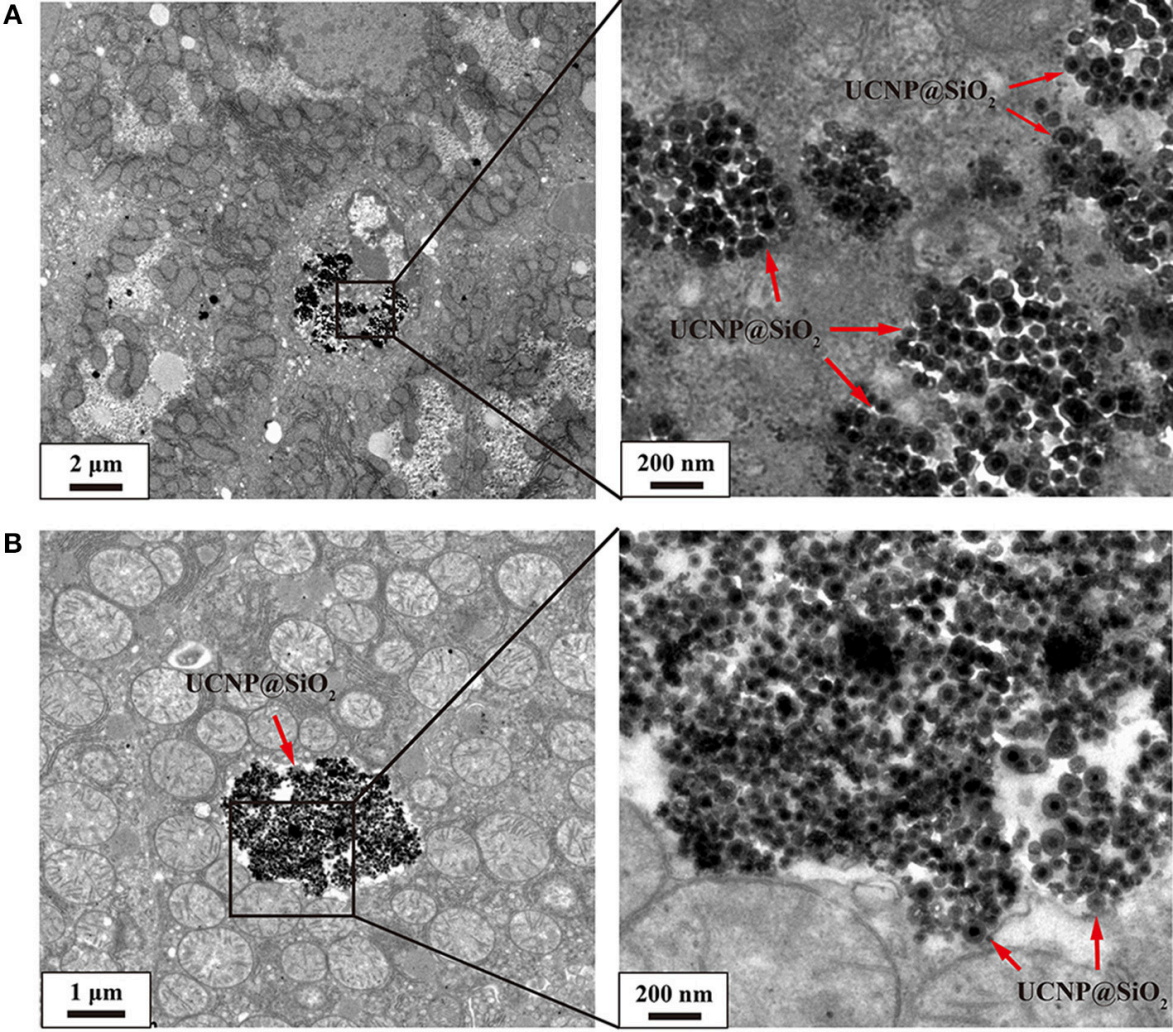
**(A)** TEM image of 20 mg/kg NaYF_4_:Yb,Er@SiO_2_ intravenously treated mouse showing aggregations of these nanoparticles (red arrow) located outside of hepatocytes. **(B)** TEM image shows aggregations of NaYF_4_:Yb,Er@SiO_2_ nanoparticles (red arrow) in the cytoplasm. UCNP@SiO_2_ denotes NaYF_4_:Yb,Er@SiO_2_.

For intravenous administration, nanoparticles show a tendency to accumulate in the liver after entry into the bloodstream. As shown in Figure 4, a large amount of NaYF_4_:Yb,Er@SiO_2_ was observed in liver tissue at day 1 and 7 after mice were intravenously injected with these nanoparticles with dose of 20 mg/kg. This result suggests that the nanoparticles were internalized by hepatocytes.

In order to further evaluate the bioavailability of NaYF_4_:Yb,Er@SiO_2_ nanoparticles, we measured the contents of Y^3+^ in blood. For the oral administration group, the contents of Y^3+^ cannot be detected in blood after 7 and 14 days consecutive oral exposure (Figure S9). By comparison, relatively high concentration of Y^3+^ has been observed in blood after intravenous injection of these nanoparticles. This can be ascribed to the low bioavailability of NaYF_4_:Yb/Er@SiO_2_ nanoparticles via gavage administration route, which is consistent with the results of ultramicrostructure observation of Peyer's patch.

### Body Weight and Organ Index of Mice

**Figure 5:**
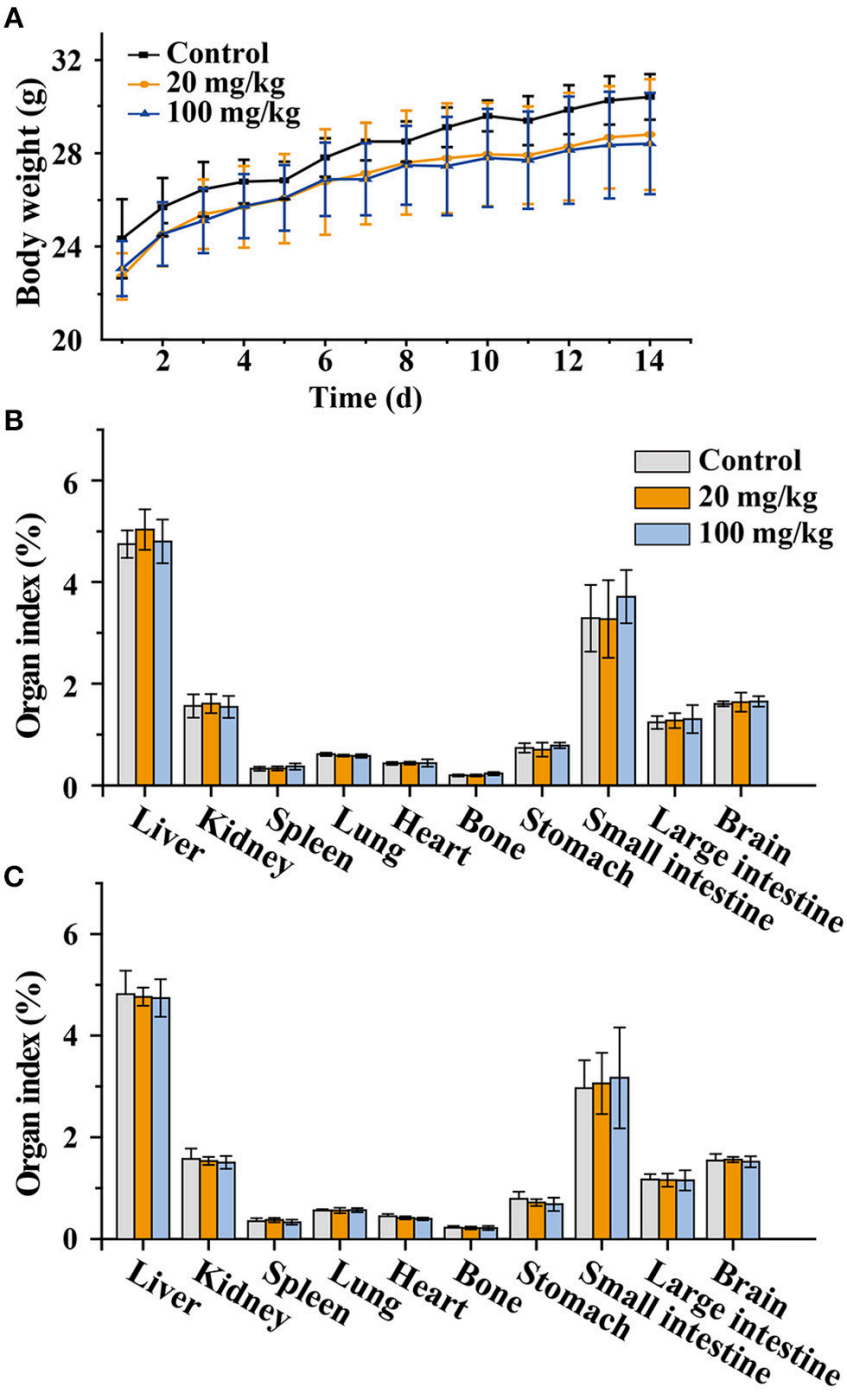
**(A)** Weight changes in animals after continued administration for 14 days (*n* = 6). **(B,C)** Organ index changes in animals after consecutive administration for 7 **(B)** and 14 **(C)** days (*n* = 6).

To evaluate the toxic effects of NaYF_4_:Yb,Er@SiO_2_ in mice, the body weights of mice were recorded every day during consecutive gavage administration. As shown in Figure 5A, death, obvious body weight decrease, and other signs of significant weakness were not observed in mice treated with NaYF_4_:Yb,Er@SiO_2_ over the 14-day period. The body weights of the NaYF_4_:Yb,Er@SiO_2_ treated groups increased in a pattern similar to that of the control group. Organ index is a key parameter in toxicity evaluation. Our results showed that there was no difference between the treatment group and the control group after gavage administration of NaYF_4_:Yb,Er@SiO_2_ either at a dose of 20 or 100 mg/kg (Figures 5B,C). These results demonstrate that NaYF_4_:Yb,Er@SiO_2_ nanoparticles have no effect on the body weight and organ index of mice.

### Histological Analysis

**Figure 6:**
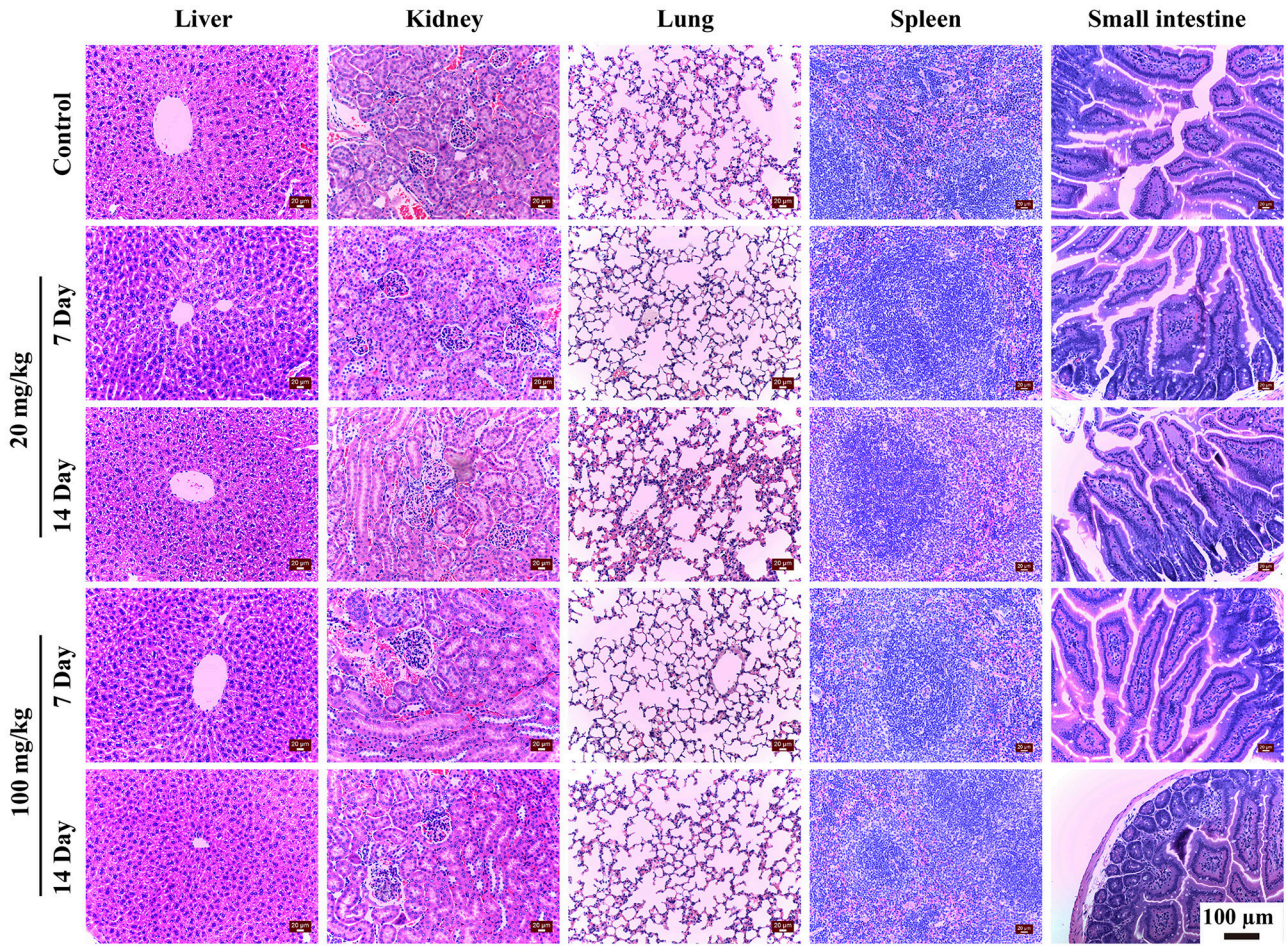
Histopathological observation of liver, kidney, lung, spleen and small intestine of mice after 7 and 14 days' consecutive gavage administration of NaYF_4_:Yb,Er@SiO_2_ at different doses.

Histological analysis of vital organs is important to evaluate whether NaYF_4_:Yb,Er@SiO_2_ could cause tissue damage, inflammation, or lesions. The analysis was performed on the liver, kidney, lung, spleen, and small intestine to investigate signs of the potential toxicity of NaYF_4_:Yb,Er@SiO_2_ at doses of 20 and 100 mg/kg for 7 and 14 days. As shown in Figure 6, hepatocytes were arranged in rows that radiate out from the central vein, and no inflammatory infiltrates of hepatocytes was observed in the liver samples (the first column). There was no change in the morphology of the renal corpuscles and renal tubules in the experimental and control groups (the second column). The glomerular structure was easy to distinguish and there was no sign of inflammatory infiltrates and necrosis. In addition, no pulmonary fibrosis or other abnormal phenomena was observed in the lung tissues for experimental groups (the third column). The white pulp and red pulp have normal appearance in spleen tissues (the forth column). The experimental and control mice showed normal intestine villi (the fifth column). Furthermore, the shape of the small intestine was normal, the tissue was intact, no inflammatory cells were infiltrated, and no bleeding was observed. In addition, we also conducted histological analysis on major organs (liver, kidney, lung, and spleen) after intravenous injection of NaYF_4_:Yb,Er@SiO_2_ nanoparticles into mice for 1 day. As shown in Figure S10, there was no obvious sign of abnormality in these major organs. Our results are also consistent with the previous study involving polyethyleneimine modified NaYF_4_:Yb,Er nanoparticles (Yu et al., 2017). In all, these results indicate that there are no obvious difference of these organs between the experimental group and control group, and the organs of experimental group exhibited healthy structural features.

### Effect of Gavage Exposure to NaYF_4_:Yb,Er@SiO_2_ on the Distribution of Zinc and Copper in Mice

The absorption of certain metal element may affect levels of essential metal elements in animals. Zinc plays a critical role in many biological functions including antioxidant defense, cell signaling, and gene expression. Copper is vital and essential to the proper functioning of organs and metabolic processes. Like all essential elements and nutrients, copper excess, or deficiency has adverse health effects.

**Figure 7:**
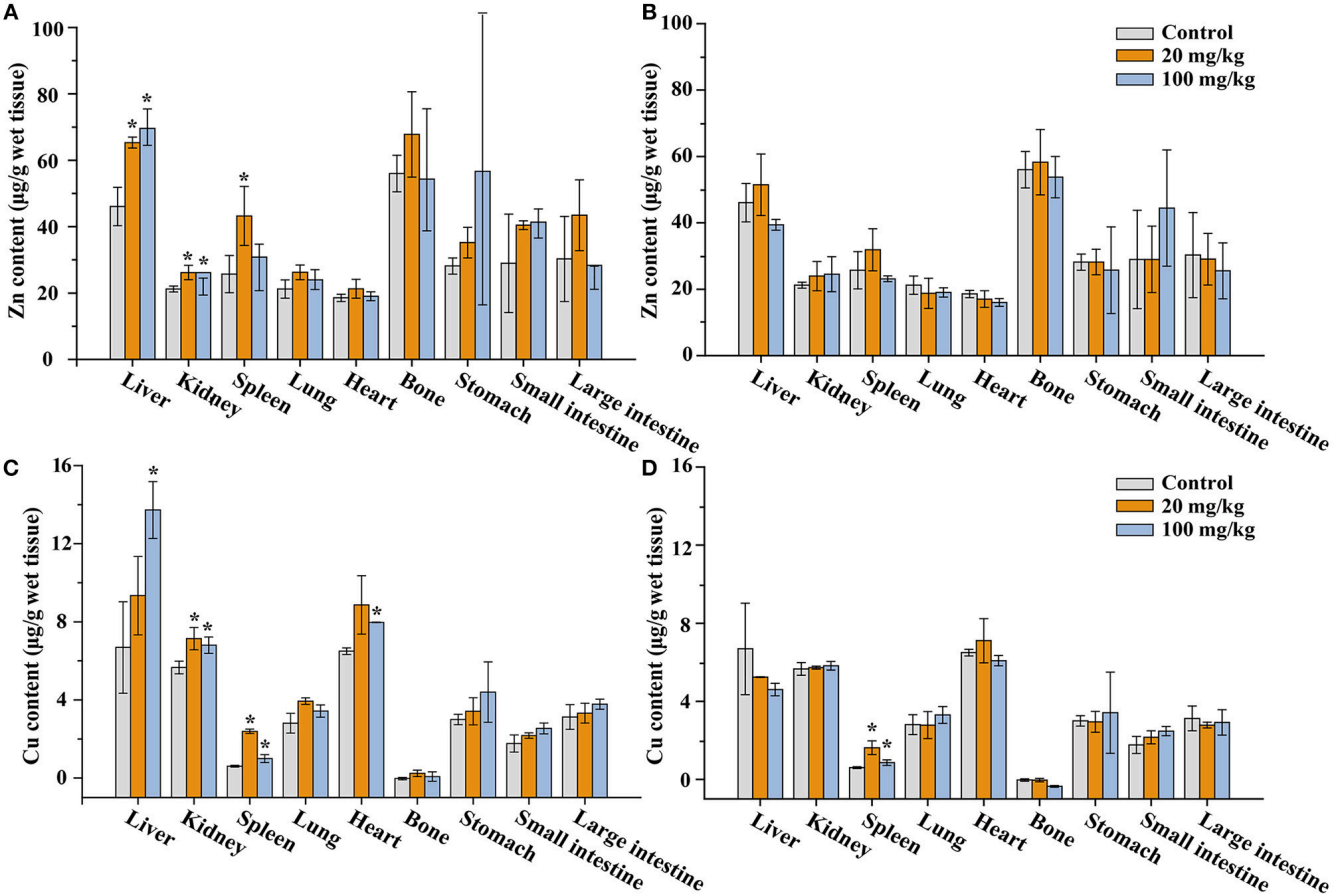
Zinc **(A,B)** and copper **(C,D)** content in tissues of the mice after 7 **(A,C)** and 14 days' **(B,D)** consecutive gavage administration of NaYF_4_:Yb,Er@SiO_2_, respectively. *Significant difference vs. the corresponding control (*P* < 0.05, *n* = 3).

To evaluate the influence of NaYF_4_:Yb,Er@SiO_2_ exposure, the contents of trace essential elements Zn and Cu in tissues in NaYF_4_:Yb,Er@SiO_2_-treated mice were measured by ICP-MS. Zinc levels in different organs of mice were shown in Figures 7A,B. After 7 days post-administration, we found that Zn concentrations significantly increase in spleen at doses of 20 mg/kg of NaYF_4_:Yb,Er@SiO_2_, and they significantly changed in liver, kidney at both doses of 20 and 100 mg/kg. However, the Zn level went back to the control level after 14-day consecutive oral exposure. In addition, copper contents in liver significantly increased at the dose of 100 mg/kg of NaYF_4_:Yb,Er@SiO_2_, while they significantly increased in kidneys, spleen and heart at both doses of 20 and 100 mg/kg NaYF_4_:Yb,Er@SiO_2_ (Figure 7C). Although the Cu level in spleen didn't go back to the control level, they reduced to the control level in liver, and kidney (Figure 7D). In addition, Zn level only significantly decreased in bone at day 1 after intravenously administration of NaYF_4_:Yb,Er@SiO_2_ at the dose of 20 mg/kg (Figure S11A). By contrast, Cu levels significantly increased in bone at day 1, and they significantly increased in liver, spleen, lung, heart, and stomach at day 7 (Figure S11B). We didn't observe the recovery in Cu level, maybe because the Cu level in mice need to take longer time to recover (i.e., 28 days) as selenium did in a previous report (Tang et al., 2016). Taken together, these results suggest that NaYF_4_:Yb,Er@SiO_2_ could slightly change the zinc and copper level of certain organs in mice. However, these changes can be recovered after a period of time.

### Serum Biochemical Analyses

Serum biochemical analysis is usually used to determine whether the function of vital organs is damaged. Liver function parameters including alanine aminotransferase (ALT, IU/L), aspartate aminotransferase (AST, IU/L), alkaline phosphatase (ALP, IU/L) and kidney index blood urea nitrogen (BUN, mmol/L), and creatine (Crea mmol/L) were measured. Results showed that ALT, AST and ALP remain unchanged after consecutive administration for 7 and 14 days (Table S2). The BUN levels in nanoparticles treatment groups were significantly elevated at day 7, while they came back to the normal level at day 14. The Crea level in the 100 mg/kg NaYF_4_:Yb,Er@SiO_2_ group significantly declined compared with the control at day 14. In brief, NaYF_4_:Yb,Er@SiO_2_ nanoparticles induce slight fluctuations in BUN and Crea levels after oral administration, but the levels remain in a normal range (Lu et al., 2010).

**Figure 8:**
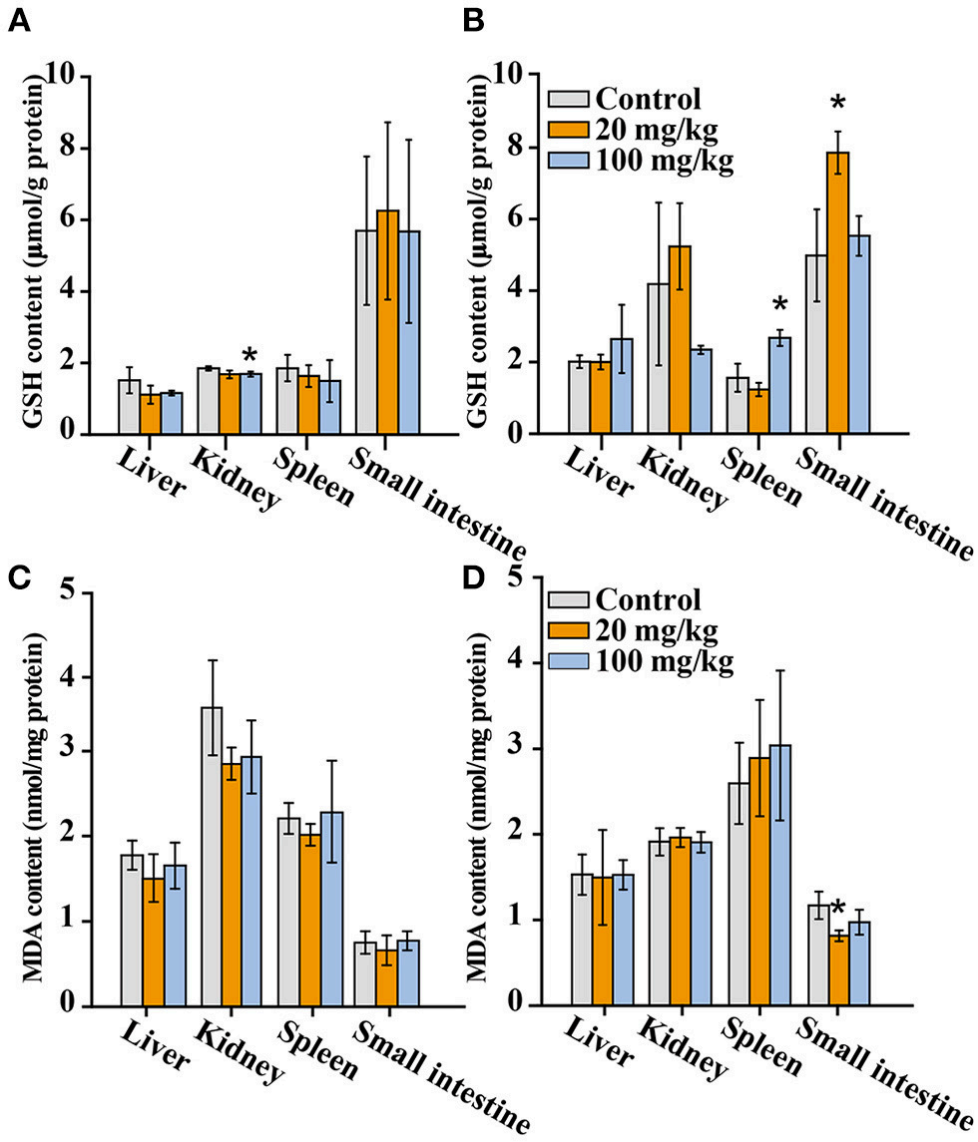
GSH **(A,B)** and MDA **(C,D)** levels in main organs of mice after consecutive gavage administration of NaYF_4_:Yb,Er@SiO_2_ (*n* = 4). **(A,C)**: 7 days; **(B,D)**: 14 days. *Significant difference vs. the corresponding control (*P* < 0.05).

The oxidative stress is an important cause of injury or inflammation for certain organs. Therefore, we further measured the levels of glutathione (GSH) and malondialdehyde (MDA) in liver, kidneys, spleen, and small intestine. As shown in Figure 8, The GSH level was the highest in small intestine, reaching a value of 6 μmol/g protein, while the level in liver, kidneys, and spleen was about 2 μmol/g protein. The GSH levels in kidney (day 7), spleen and small intestine (day 14) of the 100 and 20 mg/kg NaYF_4_:Yb,Er@SiO_2_-treated group were significantly different from that of corresponding control, respectively. The MDA level in NaYF_4_:Yb,Er@SiO_2_-treated group did not show any difference with the control at day 7 after gavage administration. However, the MDA level exhibit significant difference in small intestine at day 14 with control after gavage administration of nanoparticles. In brief, it can be concluded that NaYF_4_:Yb,Er@SiO_2_ just induce slight fluctuations of GSH and MDA levels in certain organs.

**Figure 9:**
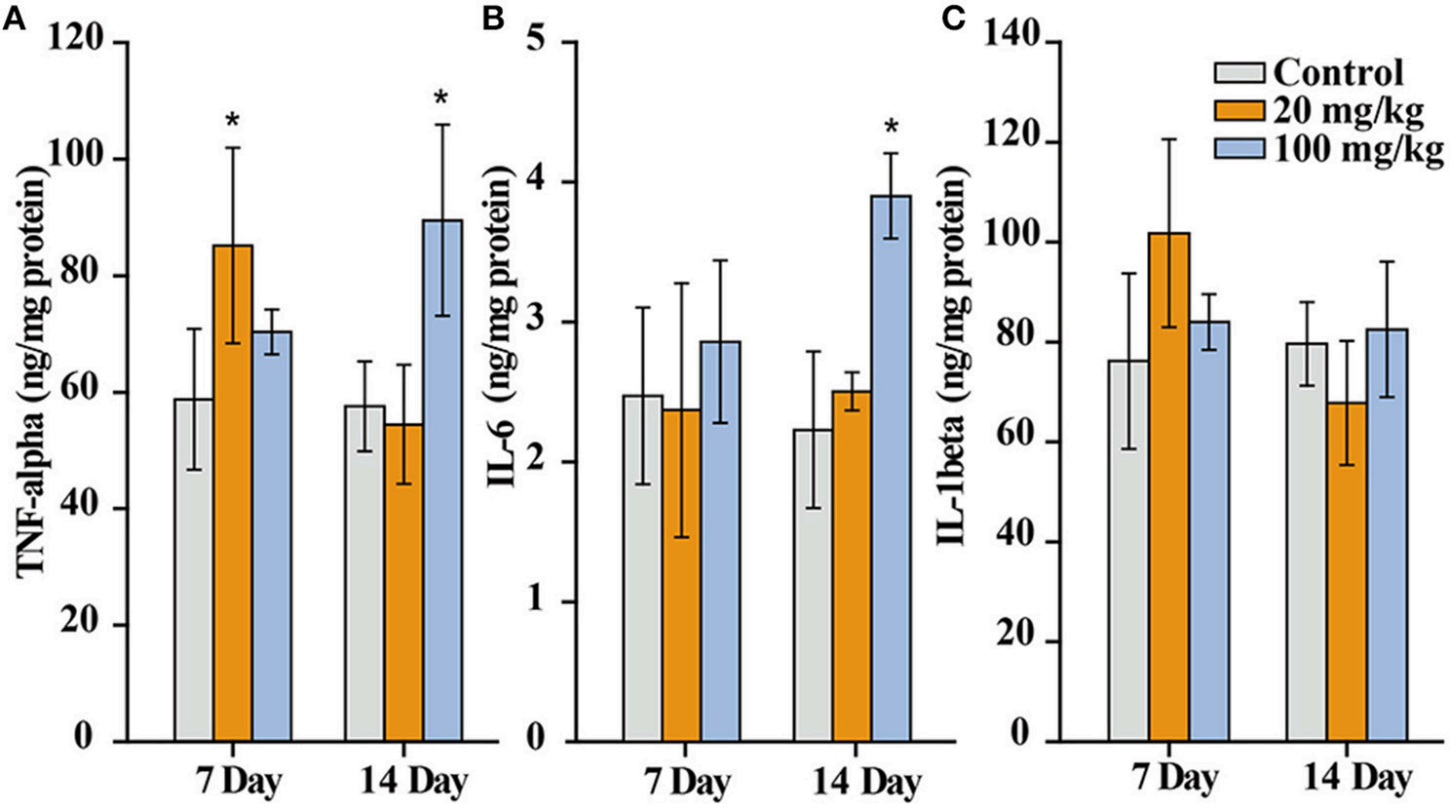
TNF-α **(A)**, IL-6 **(B)**, and IL-1β **(C)** level in liver of mice after 7 and 14 days consecutive gavage administration of NaYF_4_:Yb,Er@SiO_2_ (*n* = 5). *Significant difference vs. the corresponding control (*P* < 0.05).

Cytokines are secreted by inflammatory cells, which are involved in the immune response of the organism to foreign nanoparticles. This is the pathological basis of the occurrence and development of tissue injury. Particularly, tumor necrosis factor-alpha (TNF-alpha) is involved in inflammation and immune response. Interleukin-6 (IL-6) is involved in the pathophysiological processes of various inflammatory diseases. Interleukin-1 beta (IL-1beta) can stimulate other cytokines or inflammatory mediators, inducing the expression of immune molecules. Therefore, we detected the expression of TNF-alpha, IL-6, and IL-1beta in the liver to observe whether orally administrated NaYF_4_:Yb,Er@SiO_2_ could induce inflammation in the liver of mice. As shown in Figure 9, we found TNF-alpha and IL-6 are elevated in the NaYF_4_:Yb,Er@SiO_2_ (100 mg/kg) treated group compared with the control at day 14 after gavage administration, and significant difference of TNF-alpha only is observed in the 20 mg/kg treatment group at day 7. In addition, there was no difference of IL-1beta between the nanoparticles treated group and the control. Therefore, NaYF_4_:Yb,Er@SiO_2_ nanoparticles change the level of TNF-α and IL-6, while there is no influence to IL-1β after consecutive administration.

## Conclusions

In this study, we systematically investigate the bioavailability, biodistribution, and toxicity of orally administered NaYF_4_:Yb,Er@SiO_2_ nanoparticles with an average diameter of 50 nm. Our results show that these nanoparticles can be absorbed by mice with oral administration and the intestinal absorption through Peyer's patch was confirmed by TEM measurement. In addition, we demonstrate that the nanoparticles with intravenous injection are trapped in hepatocytes. The biodistribution of NaYF_4_:Yb,Er@SiO_2_ is particularly dependent on the administration routes. Specifically, NaYF_4_:Yb,Er@SiO_2_ nanoparticles mainly accumulate in bone, stomach, and intestine by oral administration, while these nanoparticles mainly accumulate in liver and spleen by intravenous administration. Furthermore, our results suggest that there is no overt toxicity of NaYF_4_:Yb,Er@SiO_2_ in mice even after consecutive oral exposure for 14 days at a high dose of 100 mg/kg. Collectively, these results provide an important reference for the future medical and clinical applications of inorganic nanoparticles.

## Author Contributions

MZ, XG, D-MK, HT, and J-ZZ performed the experiments. HW, BG, and MC analyzed the data. QS and LS contributed to design of the experiments and write the manuscript. All the authors discussed the results and revised the manuscript.

### Conflict of Interest Statement

The authors declare that the research was conducted in the absence of any commercial or financial relationships that could be construed as a potential conflict of interest.

## References

[B1] Abdul J. R. Zhang Y. (2008). Biocompatibility of silica coated NaYF_4_ upconversion fluorescent nanocrystals. Biomaterials 29, 4122–4128. 10.1016/j.biomaterials.2008.07.012 18675453

[B2] Auzel F. (2004). Upconversion and anti-stokes processes with f and d ions in solids. Chem. Rev. 35, 139–173. 10.1021/cr020357g 14719973

[B3] Bettinelli M. Carlos L. Liu X. (2015). Lanthanide-doped upconversion nanoparticles. Phys. Today 68, 38–44. 10.1063/pt.3.2913

[B4] Chen B. Su Q. Kong W. Wang Y. Shi P. Wang F. (2018). Energy transfer-based biodetection using optical nanomaterials. J. Mater. Chem. B 6, 2924–2944. 10.1039/c8tb00614h 32254330

[B5] Chen G. Qiu H. Prasad P. N. Chen X. (2014). Up-conversion nanoparticles: design, nanochemistry, and applications in theranostics. Chem. Rev. 114, 5161–5214. 10.1021/cr400425h 24605868 PMC4039352

[B6] Chen N. Song Z. M. Tang H. Xi W. S. Cao A. Liu Y.. (2016). Toxicological effects of Caco-2 cells following short-term and long-term exposure to Ag nanoparticles. Int. J. Mol. Sci. 17:974. 10.3390/ijms17060974 27338357 PMC4926506

[B7] Cheng L. Yang K. Shao M. Lu X. Liu Z. (2011). *In vivo* pharmacokinetics, long-term biodistribution and toxicology study of functionalized upconversion nanoparticles in mice. Nanomedicine 6, 1327–1340. 10.2217/NNM.11.56 21834646

[B8] Gao J. Li R. Wang F. Liu X. Zhang J. Hu L.. (2017). Determining the cytotoxicity of rare earth element nanoparticles in macrophages and the involvement of membrane damage. Environ. Sci. Technol. 51, 13938–13948. 10.1021/acs.est.7b04231 29121463

[B9] Gardner M. J. Altman D. G. (1986). Confidence intervals rather than P values: estimation rather than hypothesis testing. Br. Med. J. 292, 746–750. 10.1136/bmj.292.6522.746 3082422 PMC1339793

[B10] Gnach A. Lipinski T. Bednarkiewicz A. Rybka J. Capobianco J. A. (2015). Upconverting nanoparticles: assessing the toxicity. Chem. Soc. Rev. 44, 1561–1584. 10.1039/c4cs00177j 25176037

[B11] Green K. Huang K. Pan H. Han G. Lim S. F. (2018). Optical temperature sensing with infrared excited upconversion nanoparticles. Front. Chem. 6:416. 10.3389/fchem.2018.00416 30320058 PMC6166686

[B12] Jalani G. Tam V. Vetrone F. Cerruti M. (2018). Seeing, targeting and delivering with upconverting nanoparticles. J. Am. Chem. Soc. 140, 10923–10931. 10.1021/jacs.8b03977 30113851

[B13] Jang G. H. Hwang M. P. Kim S. Y. Jang H. S. Lee K. H. (2014). A systematic *in-vivo* toxicity evaluation of nanophosphor particles via zebrafish models. Biomaterials 35, 440–449. 10.1016/j.biomaterials.2013.09.054 24094937

[B14] Li R. Ji Z. Dong J. Chang C. H. Wang X. Sun B.. (2015a). Enhancing the imaging and biosafety of upconversion nanoparticles through phosphonate coating. ACS Nano 9, 3293–3306. 10.1021/acsnano.5b00439 25727446 PMC4415359

[B15] Li X. Zhang F. Zhao D. (2015b). Lab on upconversion nanoparticles: optical properties and applications engineering via designed nanostructure. Chem. Soc. Rev. 44, 1346–1378. 10.1039/c4cs00163j 25052250

[B16] Li Z. Yuan H. Yuan W. Su Q. Li F. (2017). Upconversion nanoprobes for biodetections. Coord. Chem. Rev. 354, 155–168. 10.1016/j.ccr.2017.06.025

[B17] Lim S. F. Riehn R. Ryu W. S. Khanarian N. Tung C. K. Tank D.. (2006). *In vivo* and scanning electron microscopy imaging of upconverting nanophosphors in *Caenorhabditis* elegans. Nano Lett. 6, 169–174. 10.1021/nl0519175 16464029

[B18] Liu J. N. Bu W. B. Shi J. L. (2015). Silica coated upconversion nanoparticles: a versatile platform for the development of efficient theranostics. Acc. Chem. Res. 48, 1797–1805. 10.1021/acs.accounts.5b00078 26057000

[B19] Liu L. Wang S. Zhao B. Pei P. Fan Y. Li X.. (2018). Er^3+^ sensitized 1530 nm to 1180 nm second near-infrared window upconversion nanocrystals for *in vivo* biosensing. Angew. Chem. Int. Ed. 57, 7518–7522. 10.1002/anie.201802889 29719100

[B20] Liu Y. Zhou S. Zhuo Z. Li R. Chen Z. Hong M.. (2016). *In vitro* upconverting/downshifting luminescent detection of tumor markers based on Eu^3+^-activated core-shell-shell lanthanide nanoprobes. Chem. Sci. 7, 5013–5019. 10.1039/c6sc01195k 30155152 PMC6018526

[B21] Liu Z. Ju E. Liu J. Du Y. Li Z. Yuan Q.. (2013). Direct visualization of gastrointestinal tract with lanthanide-doped BaYbF_5_ upconversion nanoprobes. Biomaterials 34, 7444–7452. 10.1016/j.biomaterials.2013.06.060 23849344

[B22] Liu Z. Li Z. Liu J. Gu S. Yuan Q. Ren J.. (2012). Long-circulating Er^3+^-doped Yb_2_O_3_ up-conversion nanoparticle as an *in vivo* X-ray CT imaging contrast agent. Biomaterials 33, 6748–6757. 10.1016/j.biomaterials.2012.06.033 22770569

[B23] Loginova Y. F. Dezhurov S. V. Zherdeva V. V. Kazachkina N. I. Wakstein M. S. Savitsky A. P. (2012). Biodistribution and stability of CdSe core quantum dots in mouse digestive tract following per os administration: advantages of double polymer/silica coated nanocrystals. Biochem. Bioph. Res. Commun. 419, 54–59. 10.1016/j.bbrc.2012.01.123 22321397

[B24] Lu J. Liong M. Li Z. Zink J. I. Tamanoi F. (2010). Biocompatibility, biodistribution, and drug-delivery efficiency of mesoporous silica nanoparticles for cancer therapy in animals. Small 6, 1794–1805. 10.1002/smll.201000538 20623530 PMC2952648

[B25] Lu Y. Zhao J. Zhang R. Liu Y. Liu D. Goldys E. M. (2013). Tunable lifetime multiplexing using luminescent nanocrystals. Nat. Photonics 8, 32–36. 10.1038/nphoton.2013.322

[B26] Lucky S. S. Idris N. M. Huang K. Kim J. Li Z. Thong P. S.. (2016). *In vivo* biocompatibility, biodistribution and therapeutic efficiency of titania coated upconversion nanoparticles for photodynamic therapy of solid oral cancers. Theranostics 6, 1844–1865. 10.7150/thno.15088 27570555 PMC4997241

[B27] Lundquist P. Artursson P. (2016). Oral absorption of peptides and nanoparticles across the human intestine: opportunities, limitations and studies in human tissues. Adv. Drug Delivery Rev. 106, 256–276. 10.1016/j.addr.2016.07.007 27496705

[B28] Ortgies D. H. Tan M. Ximendes E. C. Del Rosal B. Hu J. Xu L.. (2018). Lifetime-encoded infrared-emitting nanoparticles for *in vivo* multiplexed imaging. ACS Nano 12, 4362–4368. 10.1021/acsnano.7b09189 29697971

[B29] Peng J. Sun Y. Liu Q. Yang Y. Zhou J. Feng W. (2012). Upconversion nanoparticles dramatically promote plant growth without toxicity. Nano Res. 5, 770–782. 10.1007/s12274-012-0261-y

[B30] Plohl O. Kralj S. Majaron B. Frohlich E. Ponikvar-Svet M. Makovec D.. (2017). Amphiphilic coatings for the protection of upconverting nanoparticles against dissolution in aqueous media. Dalton Trans. 46, 6975–6984. 10.1039/c7dt00529f 28513723

[B31] Ramirez-Garcia G. Gutierrez-Granados S. Gallegos-Corona M. A. Palma-Tirado L. d'Orlye F. Varenne A.. (2017). Long-term toxicological effects of persistent luminescence nanoparticles after intravenous injection in mice. Int. J. Pharmaceut. 532, 686–695. 10.1016/j.ijpharm.2017.07.015 28705622

[B32] Sedlmeiera A. Gorris H. H. (2015). Surface modification and characterization of photon-upconverting nanoparticles for bioanalytical applications. Chem. Soc. Rev. 44, 1526–1560. 10.1039/c4cs00186a 25176175

[B33] Su Q. Feng W. Yang D. Li F. (2017). Resonance energy transfer in upconversion nanoplatforms for selective biodetection. Acc. Chem. Res. 50, 32–40. 10.1021/acs.accounts.6b00382 27983801

[B34] Sun S. K. Wang H. F. Yan X. P. (2018). Engineering persistent luminescence nanoparticles for biological applications: from biosensing/bioimaging to theranostics. Acc. Chem. Res. 51, 1131–1143. 10.1021/acs.accounts.7b00619 29664602

[B35] Tadashi K. Hiroaki T. (2006). How useful is SBF in predicting *in vivo* bone bioactivity? Biomaterials 27, 2907–2915. 10.1016/j.biomaterials.2006.01.017 16448693

[B36] Tang H. Yang S. T. Yang Y. F. Ke D. M. Liu J. H. Chen X.. (2016). Blood clearance, distribution, transformation, excretion, and toxicity of near-infrared quantum dots Ag_2_Se in mice. ACS Appl. Mater. Interfaces 8, 17859–17869. 10.1021/acsami.6b05057 27351208

[B37] Tian J. Zeng X. Xie X. Han S. Liew O. W. Chen Y. T.. (2015). Intracellular adenosine triphosphate deprivation through lanthanide-doped nanoparticles. J. Am. Chem. Soc. 137, 6550–6558. 10.1021/jacs.5b00981 25923914

[B38] Tsai Y. C. Vijayaraghavan P. Chiang W. H. Chen H. H. Liu T. I. Shen M. Y.. (2018). Targeted delivery of functionalized upconversion nanoparticles for externally triggered photothermal/photodynamic therapies of brain glioblastoma. Theranostics 8, 1435–1448. 10.7150/thno.22482 29507632 PMC5835948

[B39] Walczak A. P. Kramer E. Hendriksen P. J. Tromp P. Helsper J. P. van der Zande M.. (2015). Translocation of differently sized and charged polystyrene nanoparticles in *in vitro* intestinal cell models of increasing complexity. Nanotoxicology 9, 453–461. 10.3109/17435390.2014.944599 25093449

[B40] Wang K. Ma J. He M. Gao G. Xu H. Sang J.. (2013). Toxicity assessments of near-infrared upconversion luminescent LaF_3_:Yb,Er in early development of zebrafish embryos. Theranostics 3, 258–266. 10.7150/thno.5701 23606912 PMC3630526

[B41] Wang Y. F. Sun L. D. Xiao J. W. Feng W. Zhou J. C. Shen J.. (2012). Rare-earth nanoparticles with enhanced upconversion emission and suppressed rare-earth-ion leakage. Chemistry 18, 5558–5564. 10.1002/chem.201103485 22488939

[B42] Wozniak A. Noculak A. Gapinski J. Kociolek D. Boś-Liedke A. Zalewski T. (2016). Cytotoxicity and imaging studies of β-NaGdF_4_:Yb^3+^Er^3+^@PEG-Mo nanorods. RSC Adv. 6, 95633–95643. 10.1039/c6ra20415e

[B43] Wu X. Hu P. Hu S. Chen Z. Yan H. Tang Z. (2016). Upconversion nanoparticles for differential imaging of plant cells and detection of fluorescent dyes. J. Rare Earth 34, 208–220. 10.1016/s1002-0721(16)60016-9

[B44] Wysokinska E. Cichos J. Ziolo E. Bednarkiewicz A. Strzadala L. Karbowiak M. (2016). Cytotoxic interactions of bare and coated NaGdF_4_:Yb^3+^:Er^3+^ *in vivo* biodistribution imaging and toxicity of polyacrylic acid-coated upconversion nanophosphors. nanoparticles with macrophage and fibroblast cells. Toxicol. In Vitro 32, 16–25. 10.1016/j.tiv.2015.11.021 26639924

[B45] Xiong L. Yang T. Yang Y. Xu C. Li F. (2010). Long-term Biomaterials 31, 7078–7085. 10.1016/j.biomaterials.2010.05.065 20619791

[B46] Yang D. Ma P. Hou Z. Cheng Z. Li C. Lin J. (2015). Current advances in lanthanide ion Ln^3+^-based upconversion nanomaterials for drug delivery. Chem. Soc. Rev. 44, 1416–1448. 10.1039/c4cs00155a 24988288

[B47] Yao M. He L. McClements D. J. Xiao H. (2015). Uptake of gold nanoparticles by intestinal epithelial cells: impact of particle size on their absorption, accumulation, and toxicity. J. Agr. Food Chem. 63, 8044–8049. 10.1021/acs.jafc.5b03242 26313743

[B48] Yoshida T. Yoshioka Y. Takahashi H. Misato K. Mori T. Hirai T.. (2014). Intestinal absorption and biological effects of orally administered amorphous silica particles. Nanoscale Res. Lett. 9, 532–532. 10.1186/1556-276X-9-532 25288919 PMC4184165

[B49] Yu J. Yin W. Peng T. Chang Y. N. Zu Y. Li J.. (2017). Biodistribution, excretion, and toxicity of polyethyleneimine modified NaYF_4_:Yb,Er upconversion nanoparticles in mice via different administration routes. Nanoscale 9, 4497–4507. 10.1039/c7nr00078b 28317980

[B50] Zhou J. C. Yang Z. L. Dong W. Tang R. J. Sun L. D. Yan C. H. (2011). Bioimaging and toxicity assessments of near-infrared upconversion luminescent NaYF_4_:Yb,Tm nanocrystals. Biomaterials 32, 9059–9067. 10.1016/j.biomaterials.2011.08.038 21880365

[B51] Zhou L. Wang R. Yao C. Li X. Wang C. Zhang X. (2015). Single-band upconversion nanoprobes for multiplexed simultaneous *in situ* molecular mapping of cancer biomarkers. Nat. Commun. 6:6938 10.1038/ncomms7938 25907226 PMC4423208

