# Supplementary material for: The Bioavailability, Biodistribution, and Toxic Effects of Silica-Coated Upconversion Nanoparticles in vivo
Source: Front Chem. 2019 Apr 10;7:218. doi: 10.3389/fchem.2019.00218 (PMC6468325; doi:10.3389/fchem.2019.00218)
Supplement: Supplementary file 1 [file Table_1.docx]

Supplementary Material

The Bioavailability, Biodistribution and Toxic Effects of Silica-coated Upconversion Nanoparticles *in Vivo*

Mingzhu Zhou^1,#^, Xiaoqian Ge^2,#^, Da-Ming Ke^1,#^, Huan Tang^3^, Jun-Zheng Zhang^1^, Matteo Calvaresi^4^, Bin Gao^5^, Lining Sun^2,^*, Qianqian Su^1,^*, Haifang Wang^1^

^1^Institute of Nanochemistry and Nanobiology, Shanghai University, Shanghai 200444, China

^2^Research Center of Nano Science and Technology, and School of Material Science and Engineering, Shanghai University, Shanghai 200444, China

^3^Beijing National Laboratory for Molecular Sciences, College of Chemistry and Molecular Engineering, Peking University, Beijing 100871, ChinaBeijing National Laboratory for Molecular Sciences, College of Chemistry and Molecular Engineering, Peking University, Beijing 100871, China

^4^Dipartimento di Chimica “G. Ciamician”, Alma Mater Studiorum – Università di Bologna, via F. Selmi 2, 40126 Bologna, Italy

^5^Cancer and Stem Cell Biology Program, Duke-NUS Medical School, 8 College Road, Singapore, 169857, Singapore

***Correspondence:** Corresponding Authors: chmsqq@shu.edu.cn (Q. Su), lnsun@shu.edu.cn (L. Sun)

^#^These authors contributed equally.

**
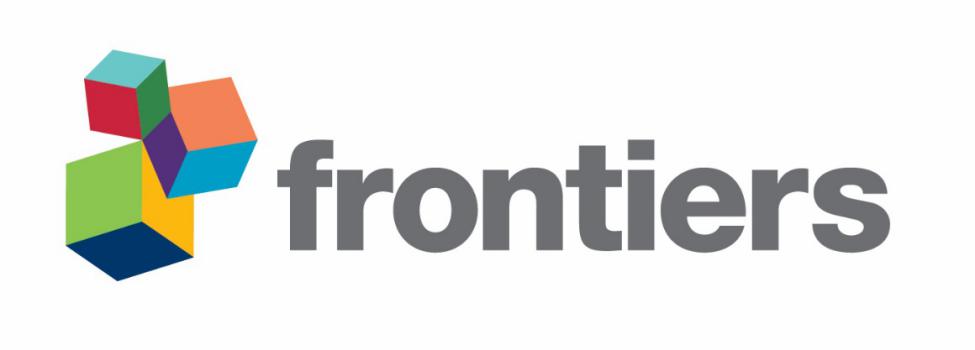
**

**Table S1.** Composition of simulated body fluid (amounts based on 1000 mL of juice) (Tang et al., 2016).

| Name | Reagent | Content |
| --- | --- | --- |
| SBF  (pH = 7.4) | NaCl | 8.035 g |
|  | NaHCO_3_ | 0.355 g |
|  | KCl | 0.225 g |
|  | K_2_HPO_4_·3H_2_O | 0.231 g |
|  | MgCl_2_·6H_2_O | 0.311 g |
|  | 1.0 M HCl | 39 mL |
|  | CaCl_2_ | 0.292 g |
|  | Na_2_SO_4_ | 0.072 g |
|  | Tris | 6.118 g |
|  | 1.0 M HCl | 0-5 mL |

Tang, H., Yang, S. T., Yang, Y. F., Ke, D. M., Liu, J. H., Chen, X., et al. (2016). Blood clearance, distribution, transformation, excretion, and toxicity of near-infrared quantum dots Ag_2_Se in mice. *ACS Appl. Mater. Interfaces* 8, 17859−17869. doi: 10.1021/acsami.6b05057.

**Table S2.** Biochemical parameters of serum in the mice after consecutive gavage administration of NaYF_4_:Yb,Er@SiO_2_. *Significant difference vs the corresponding control (P<0.05, n = 3).

| *Parameter* | 7 Day | | | 14 Day | | |
| --- | --- | --- | --- | --- | --- | --- |
|  | 0 mg/kg | 20 mg/kg | 100 mg/kg | 0 mg/kg | 20 mg/kg | 100 mg/kg |
| ALT  (IU/L) | 26.3±5.7 | 27.0±7.0 | 26. 7±4.2 | 31.3±4.2 | 33.0±7.2 | 32.0±6.2 |
| AST  (IU/L) | 108.3±22.2 | 80.3±17.9 | 113.3±29.7 | 111.0±8.7 | 91.3±18.0 | 103.3±18.1 |
| ALP  (IU/L) | 406.0±78.0 | 459.0±49.9 | 535.3±127.1 | 456.7±88.8 | 321.7±36.0 | 474.3±78.4 |
| BUN  (mmol/L) | 6.9±0.5 | 8.4±0.4* | 9.3±1.5* | 8.7±1.0 | 8.3±1.3 | 8.5±1.5 |
| Crea  (μmol/L) | 9.7±1.1 | 10.0±1.7 | 10.3±1.5 | 12.3±0.6 | 11.3±1.2 | 10.0±1.0* |

**
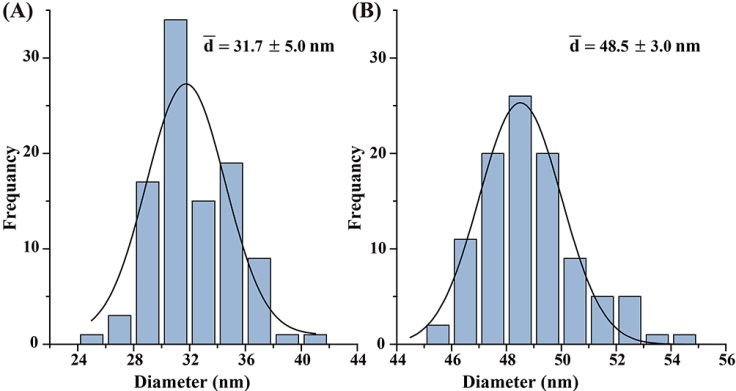
**

**Figure S1.** Size distribution of NaYF_4_:Yb,Er (A) and NaYF_4_:Yb,Er@SiO_2_ (B) nanoparticles measured by Image J software.


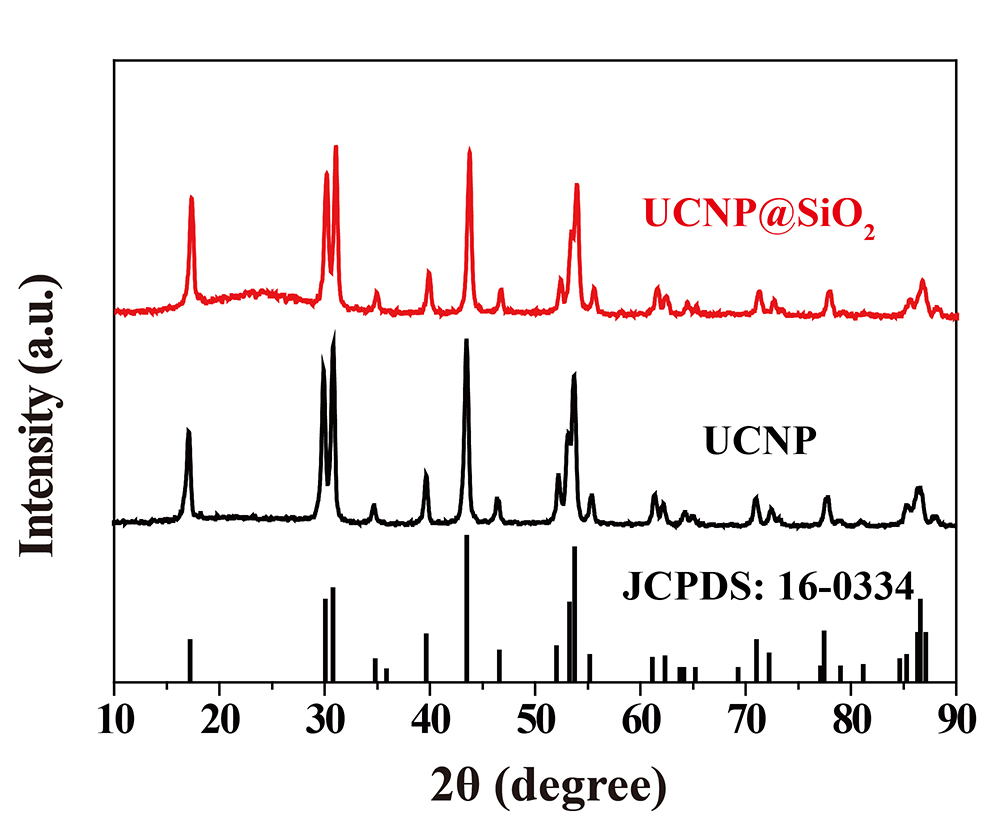


**Figure S2.** XRD patterns of NaYF_4_:Yb,Er, NaYF_4_:Yb,Er@SiO_2_, and the standard card of hexagonal NaYF_4_ (JCPDS: 16-0334). UCNP denotes NaYF_4_:Yb,Er. UCNP@SiO_2_ denotes NaYF_4_:Yb,Er@SiO_2_.


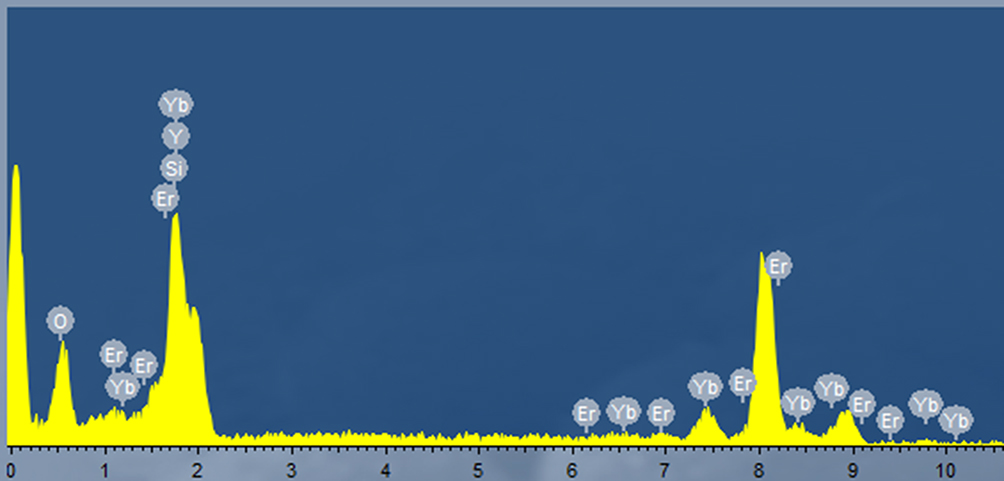


**Figure S3.** Energy dispersive X-ray (EDX) spectrum of NaYF_4_:Yb,Er@SiO_2_.


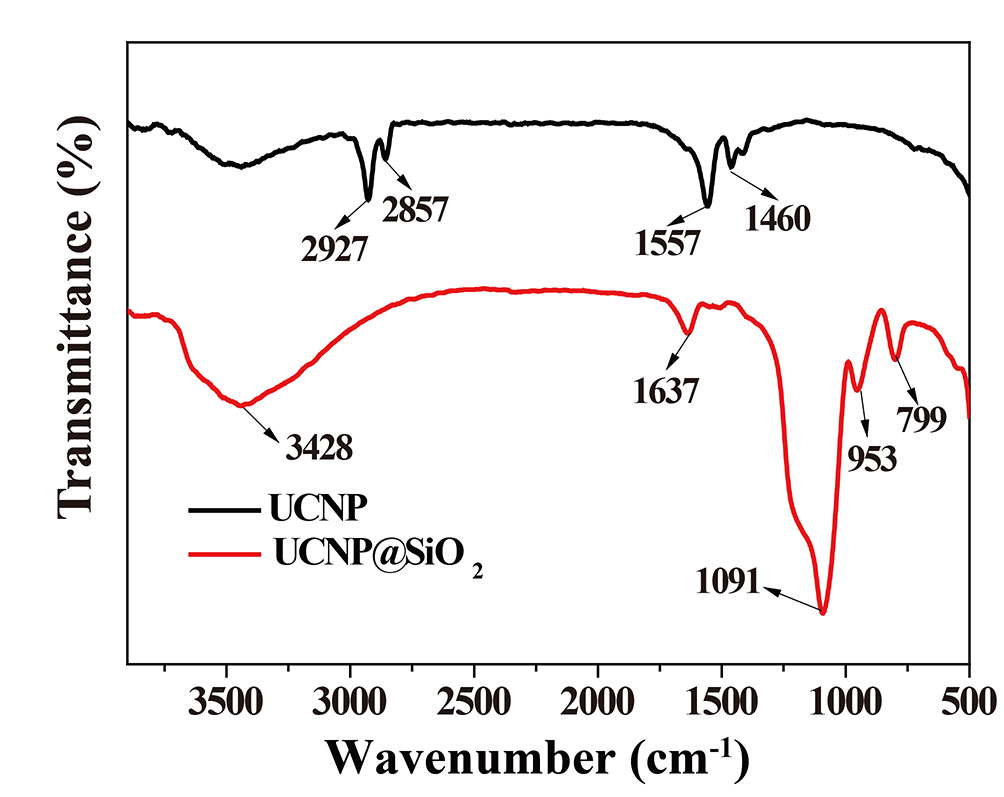


**Figure S4.** FT-IR spectra of the oleic acid coated NaYF_4_:Yb,Er and NaYF_4_:Yb,Er@SiO_2_. UCNP denotes oleic acid capped NaYF_4_:Yb,Er. UCNP@SiO_2_ denotes NaYF_4_:Yb,Er@SiO_2_.


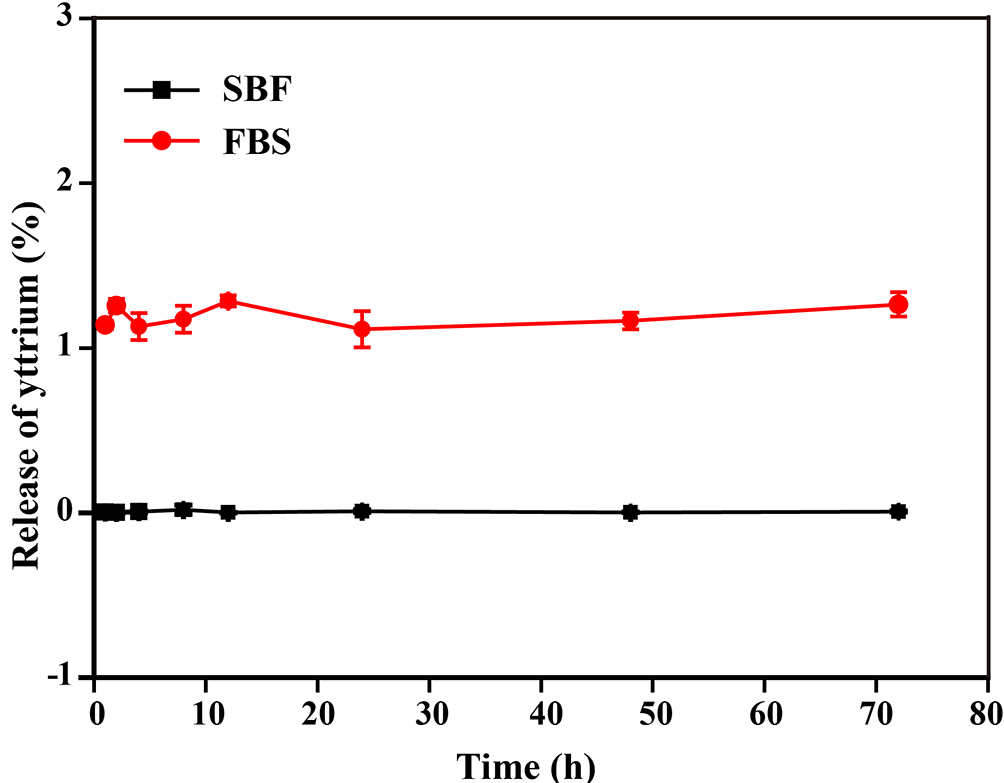


**Figure S5.** The release of yttrium from NaYF_4_:Yb,Er@SiO_2_ in SBF and FBS.

**
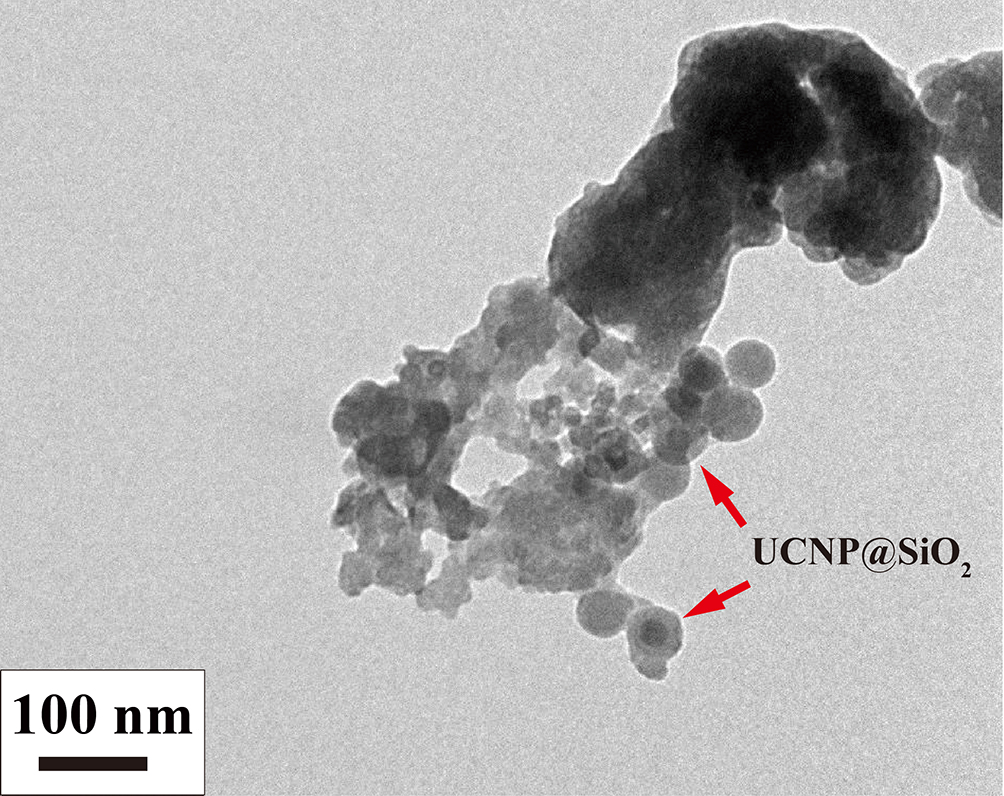
**

**Figure S6.** TEM image of NaYF_4_:Yb,Er@SiO_2_ in feces collected on the 14^th^ day after mice were administrated consecutively with NaYF_4_:Yb,Er@SiO_2_ by gavage method. UCNP@SiO_2_ denotes NaYF_4_:Yb,Er@SiO_2_.

**
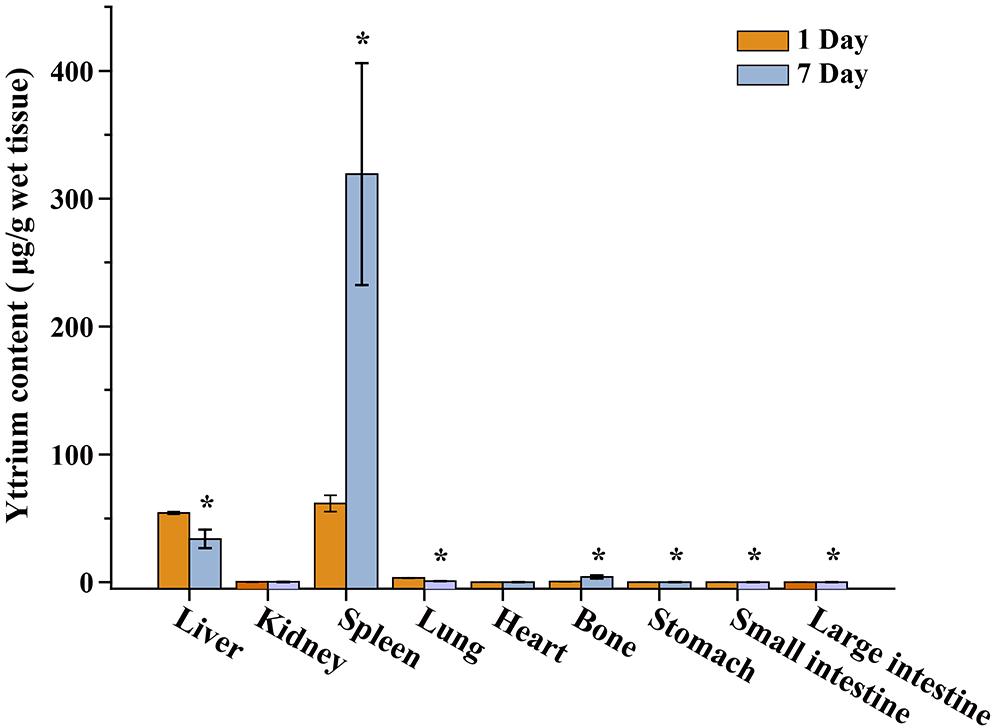
**

**Figure S7.** Yttrium content in the tissues of mice at day 1 and day 7 after a single intravenous administration of NaYF_4_:Yb,Er@SiO_2_, respectively. *Significant difference between the two treatment groups (P<0.05, n = 3).


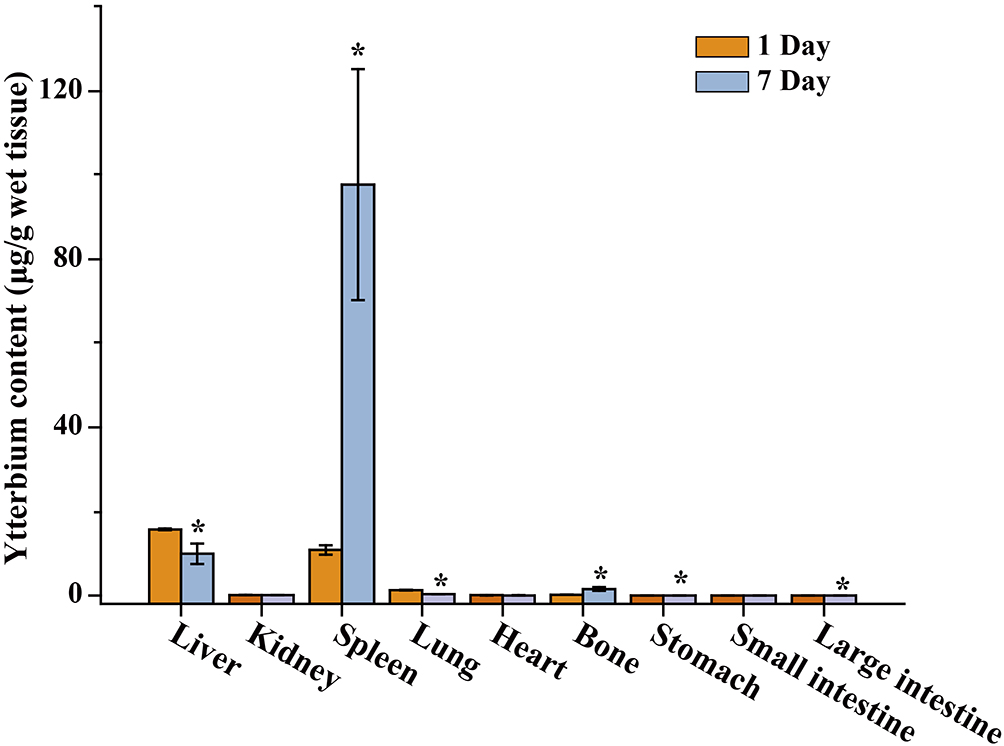


**Figure S8.** Ytterbium content in the tissues of mice at day 1 and day 7 after a single intravenous administration of NaYF_4_:Yb,Er@SiO_2_, respectively. *Significant difference between the two treatments groups (P<0.05, n = 3).

**
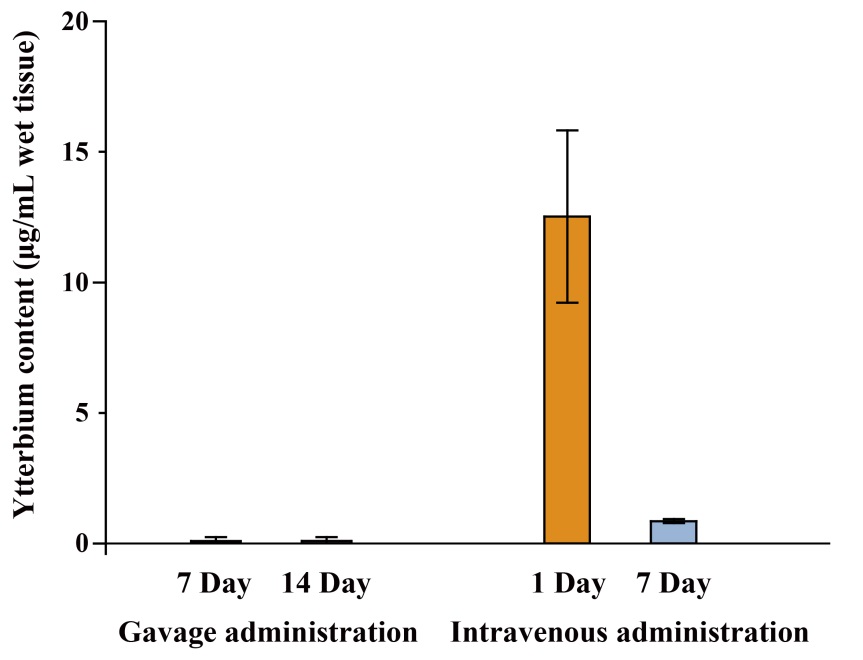
**

**Figure S9.** Ytterbium content in the blood of mice at day 7 and 14 at a dose of 20 mg/kg by gavage method and at day 1 and day 7 after a single intravenous administration of these nanoparticles with a dose of 20 mg/kg, respectively (n = 3).


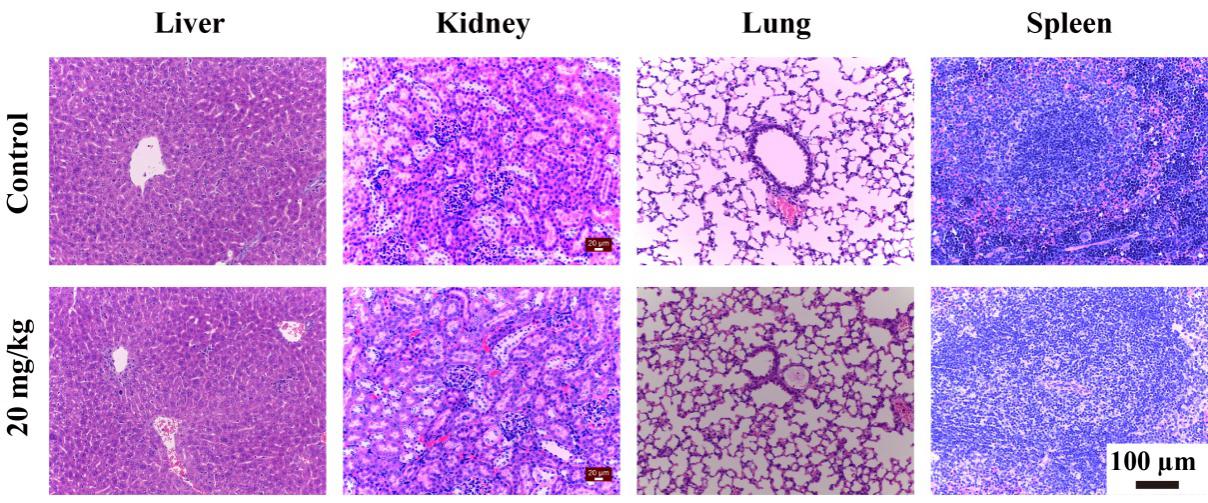


**Figure S10.** Histopathological observation of liver, kidney, lung and spleen of mice at day 1 after intravenous administration of NaYF_4_:Yb,Er@SiO_2_ into mice with a dose of 20 mg/kg.


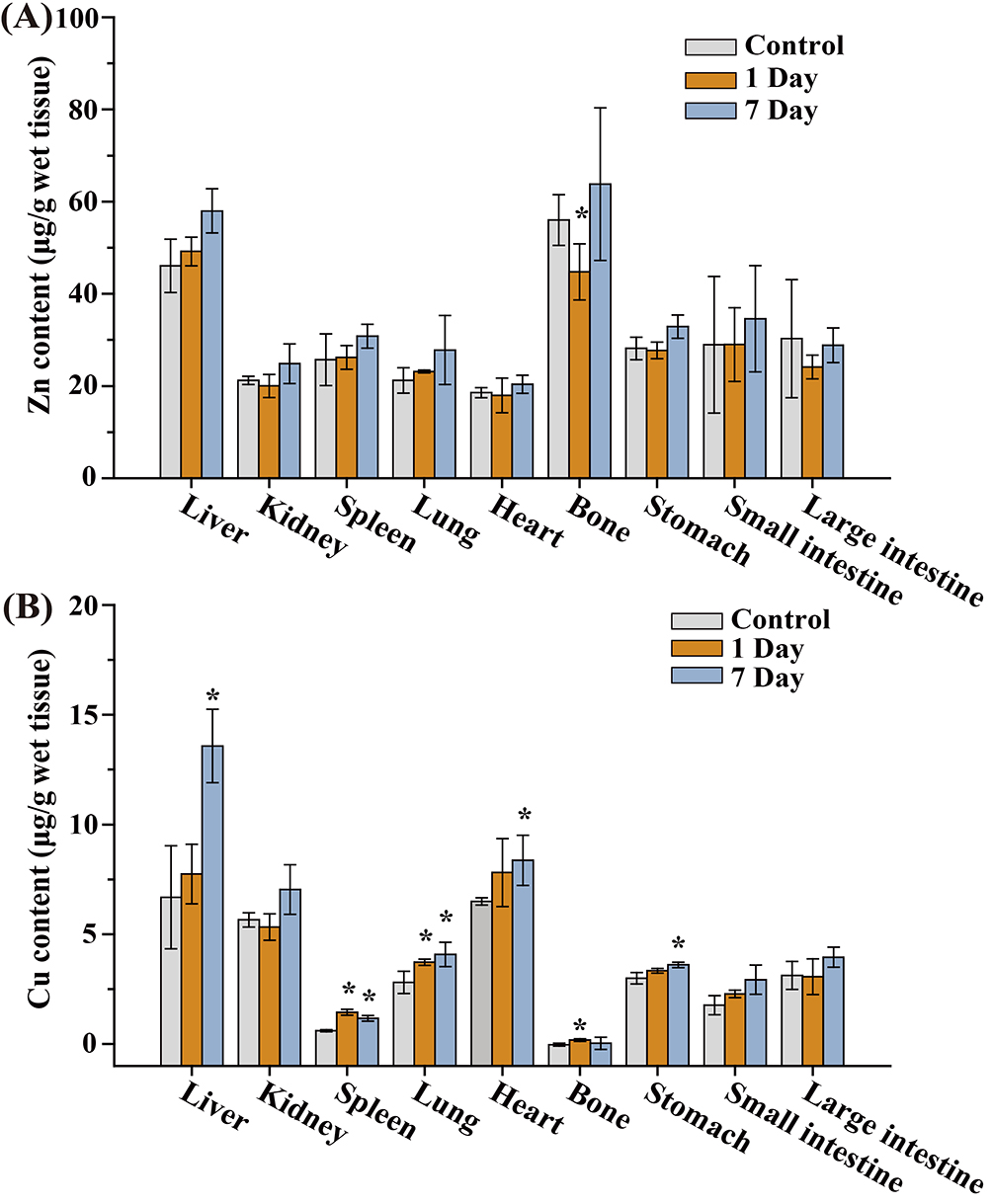


**Figure S11.** Zinc (A) and copper (B) content in the tissues of mice at day 1 and day 7 after a single intravenous administration of UCNP@SiO_2_. *Significant difference vs the corresponding control (P<0.05, n = 3).
